# Variation in auxin sensing guides AUX/IAA transcriptional repressor ubiquitylation and destruction

**DOI:** 10.1038/ncomms15706

**Published:** 2017-06-07

**Authors:** Martin Winkler, Michael Niemeyer, Antje Hellmuth, Philipp Janitza, Gideon Christ, Sophia L. Samodelov, Verona Wilde, Petra Majovsky, Marco Trujillo, Matias D. Zurbriggen, Wolfgang Hoehenwarter, Marcel Quint, Luz Irina A. Calderón Villalobos

**Affiliations:** 1 Department of Molecular Signal Processing, Leibniz Institute of Plant Biochemistry (IPB), Halle (Saale) D-06120, Germany; 2 Institute of Agricultural and Nutritional Sciences, Martin Luther University Halle-Wittenberg, Halle (Saale) D-06120, Germany; 3 Institute of Synthetic Biology, University of Düsseldorf, Düsseldorf D-40225, Germany; 4 Proteome Analytics Research Group, Leibniz Institute of Plant Biochemistry (IPB), Halle (Saale) D-06120, Germany; 5 Independent Junior Research Group Ubiquitination in Immunity, Leibniz Institute of Plant Biochemistry (IPB), Halle (Saale) D-06120, Germany; 6 Spemann Graduate School of Biology and Medicine (SGBM), University of Freiburg, Freiburg D-79104, Germany; 7 Cluster of Excellence on Plant Science (CEPLAS), University of Düsseldorf, Düsseldorf D-40225, Germany

## Abstract

Auxin is a small molecule morphogen that bridges SCF^TIR1/AFB^-AUX/IAA co-receptor interactions leading to ubiquitylation and proteasome-dependent degradation of AUX/IAA transcriptional repressors. Here, we systematically dissect auxin sensing by SCF^TIR1^-IAA6 and SCF^TIR1^-IAA19 co-receptor complexes, and assess IAA6/IAA19 ubiquitylation *in vitro* and IAA6/IAA19 degradation *in vivo.* We show that TIR1-IAA19 and TIR1-IAA6 have distinct auxin affinities that correlate with ubiquitylation and turnover dynamics of the AUX/IAA. We establish a system to track AUX/IAA ubiquitylation in IAA6 and IAA19 *in vitro* and show that it occurs in flexible hotspots in degron-flanking regions adorned with specific Lys residues. We propose that this signature is exploited during auxin-mediated SCF^TIR1^-AUX/IAA interactions. We present evidence for an evolving AUX/IAA repertoire, typified by the IAA6/IAA19 ohnologues, that discriminates the range of auxin concentrations found in plants. We postulate that the intrinsic flexibility of AUX/IAAs might bias their ubiquitylation and destruction kinetics enabling specific auxin responses.

## Abstract (web summary)

The phytohormone auxin is sensed by SCF^TIR1^-AUX/IAA receptors leading to AUX/IAA repressor ubiquitylation and turnover. Here the authors show that IAA6 and IAA19 differ in their ubiquitylation and turnover dynamics, differentially contributing to auxin sensing and enabling discrimination of auxin concentrations.

Ubiquitin-dependent dynamic turnover of transcriptional regulators via E3 ligases in response to phytohormones is pivotal for growth and development^1^,^2^,^3^,^4^,^5^. Auxin or indole-3-acetic acid (IAA) is one of the major plant regulators, and triggers extensive transcriptional reprogramming through a very short nuclear cascade^6^. Auxin drives nuclear events by modulating the recruitment of mostly short-lived AUXIN/INDOLE-3-ACETIC ACID (AUX/IAA) transcriptional repressors by multimeric SKP1/CUL1/F-Box (SCF)-type E3 ubiquitin ligases. SCF^TIR1/AFBs^ E3s control auxin-triggered molecular networks by acting at the site of auxin sensing. In a tight and regulated manner and bypassing an autocatalytic mechanism, TRANSPORT INHIBITOR RESPONSE 1 (TIR1)/AUXIN SIGNALLING F-BOX (AFB1-5) proteins assemble in an SCF^TIR1/AFBs^ complex and recruit the core degron of multifunctional AUX/IAA proteins in response to fluctuations in intracellular auxin levels^7^,^8^,^9^,^10^. By increasing the hydrophobic interactions between TIR1/AFBs and their AUX/IAA targets, auxin behaves as a molecular glue which is hereby sensed by this co-receptor system. Given the expansion of *TIR1/AFBs* and *AUX/IAA* genes in *Arabidopsis*, with six and 29 members, respectively, a broad range of auxin concentrations is likely differentially sensed via combinatorial assembly of SCF^TIR1/AFB^-AUX/IAA co-receptor complexes^11^. Through heterodimerization of their C-terminal PB1 domains^12^,^13^,^14^,^15^, AUX/IAAs interact with DNA-binding proteins of the auxin response factor (ARF) family, which specifically occupy auxin-responsive elements (AuxREs) in numerous auxin-regulated genes^16^. The primary structures of most AUX/IAAs share four regions of sequence conservation^17^ including an N-terminal domain (DI) for recruitment of transcriptional co-repressors, a core degron flanked by rate motifs^18^, and the C-terminal ubiquitin-like PB1 domain that mediates homotypic as well as heterotypic interactions (reviewed in ref. 19). AUX/IAA's inherent structural flexibility seems to allow them to accommodate different binding partners exploiting different binding modes. As AUX/IAAs are often products of early auxin-responsive genes, their repressor activity establishes robust negative feedback loops^6^,^20^. AUX/IAAs probably also undergo cyclophilin-catalysed isomerization^21^ stimulated by auxin, which facilitates recognition by SCF^TIR1/AFBs^. An increase of the nuclear auxin concentration is registered by the formation of a ternary TIR1/AFB:auxin:AUX/IAA co-receptor complex (reviewed in ref. 19). Once recruited, AUX/IAAs are predicted to be tagged with polymeric ubiquitin (Ub) chains leading them to destruction by the 26S proteasome^22^. Interestingly, an auxin-inducible degron technology has been widely utilized for conditional auxin-based depletion of proteins in various eukaryotic systems such as yeast, *Drosophila melanogaster*, *Caenorhabditis elegans* and recently mammalian cells using a combination of auxin-inducible degron tagging and CRISPR/Cas^23^,^24^. Although the core of the AUX/IAA degron located in conserved domain II (DII) is necessary for TIR1-AUX/IAA associations, it is not sufficient for full auxin-binding properties of a co-receptor *in vitro* or AUX/IAA turnover *in vivo*^11^,^18^. In fact, a bona fide AUX/IAA degron for ubiquitin-proteasome system (UPS)-mediated degradation likely consists of three elements (tripartite): the primary degron motif recognizable by cognate SCF^TIR1/AFB^-E3 ligases; a secondary degron with one (or multiple neighbouring) lysine(s) present on a ubiquitylation zone^25^; and a tertiary degron in a disordered locally flexible site located proximal to (or overlapping with) the secondary degron for engaging the proteasome^25^,^26^,^27^. Hence, rate motifs that flank the primary degron and are located in AUX/IAA-disordered regions could also modulate SCF^TIR1/AFB^-AUX/IAA interactions and AUX/IAA degradation dynamics^11^,^18^. It has been proposed that SCF^TIR1/AFB^-mediated AUX/IAA proteolysis, and the combinatorial diversity of auxin-triggered TIR1/AFB-AUX/IAA interactions build an intricate network controlling complex genetic programs^6^,^28^. The understanding of the global dynamics of auxin co-receptor assembly and its immediate impact on AUX/IAA ubiquitylation and degradation is not fully understood. Furthermore, while most studies have focused on the downstream events of auxin sensing, we lack a detailed explanation for the co-existence of the plethora of co-receptor complexes. Studies on how the SCF^TIR1/AFBs^-auxin system senses various auxin concentrations differentially targeting AUX/IAA proteins leading to their ubiquitylation and degradation are still in their infancy. Therefore, we seek to understand the evolutionary retention of AUX/IAA genes and identify paramount features that lead to SCF^TIR1^ discrimination and processing. Additionally, aiming to dissect biochemically ubiquitin conjugation of AUX/IAAs, we set to establish a tunable system to assess SCF^TIR1^-AUX/IAA assembly and specific auxin-triggered AUX/IAA ubiquitylation.

Here, we analyse inter- and intra-specific sequence variation in a selected sister pair of canonical *Arabidopsis* AUX/IAAs, *IAA6* and *IAA19*, and characterize biochemically the SCF^TIR1^-IAA6 and SCF^TIR1^-IAA19 auxin co-receptors. We also define their affinity for auxin, the kinetics of SCF^TIR1^-target assembly for these two co-receptors, and report distinct ubiquitylation patterns of IAA6 and IAA19 repressors. Ultimately, we present a model for how related proteins, that are functionally specialized to sense specific small molecule concentrations, might interpret those signals into differential stability of transcriptional regulators, regulating gene expression and developmental responses.

## Results

### IAA6 and IAA19 differ in expression and selection patterns

**Figure 1:**
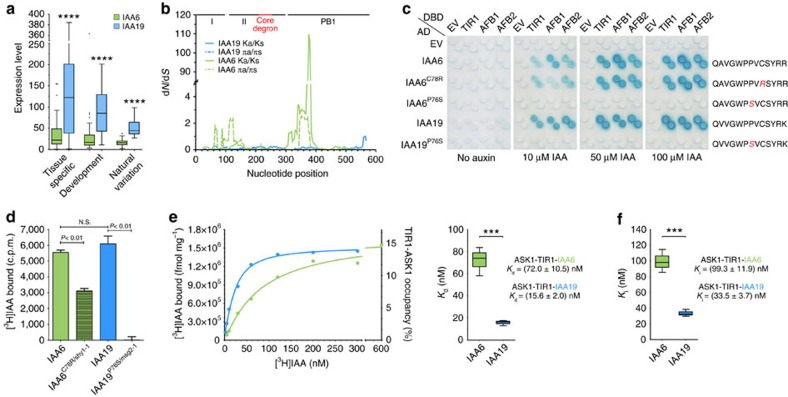
*IAA6* and *IAA19* ohnologues exhibit different selection pressure signatures and their TIR1-containing receptor complexes show dissimilar auxin binding properties. (**a**) Comparison of available *A. thaliana IAA6* (green) and *IAA19* (blue) expression data. Box plots depict IQR and the median of three different datasets obtained from the *Arabidopsis* eFP browser^78^. Outliers were defined as 1.5 × IQR. Graphs for different conditions in each data set are shown in Supplementary Fig. 2. IQR, interquartile range. (**b**) Sliding window plots of nucleotide sequence divergence between *IAA6* and *IAA19* in *A. thaliana*, *A. lyrata, A. halleri* and *C. rubella*. d*N*/d*S* ratios are plotted against the midpoint position of each 50 bp window. Black bars (top) display positions of the different protein domains. See Supplementary Data 1 for detailed AUX/IAA domain structure. (**c**) Y2H interaction matrix for TIR1, AFB1, AFB2 with IAA6, IAA19, and their putative dominant mutant versions^32^,^33^,^35^: IAA6^C78R/suppressor of hy2 (shy1-1)^, IAA6^P76S^, IAA19^P76S/massugu (msg2-1)^, carrying mutations in the degron (right). Yeast diploids containing LexA DBD-TIR1/AFBs and AD-AUX/IAAs were spotted on selective medium with increasing IAA concentrations, and β-galactosidase reporter expression indicates IAA-induced TIR1/AFB1/2-AUX/IAA interactions. EV, empty vector. (**d**) One-point saturation binding assays using 200 nM [^3^H]IAA to recombinant ASK1-TIR1-AUX/IAA ternary complexes. IAA6^C78R/shy1-1^ and IAA19^P76S/msg2-1^ mutants that mimic stabilized version of the proteins affect significantly specific auxin binding. (**e**,**f**) IAA6 and IAA19 provide ASK1-TIR1-containing complexes different auxin-sensing capabilities. (**e**) Representative saturation binding curves for IAA6 and IAA19 (left). ASK1-TIR1-IAA19 complexes bind auxin with high affinity (*K*_d_=15.6±2.00 nM), whereas IAA6-containing co-receptor complexes provide fivefold lower affinity for auxin (*K*_d_=72.0±10.5 nM) (right). (**f**) Homologous competition experiments were performed using 50 or 25 nM [^3^H]IAA for ASK1-TIR1-IAA6 or -IAA19, respectively. *IC*_50_s were obtained from curve fitting, and *K*_i_ values were calculated using the Cheng–Prusoff equation given the observed dissociations constants from saturation binding experiments. Error bars, s.d. (**d**) or minimum and maximum values (**e**,**f**) of three independent biological replicates. Asterisks denote significant statistical differences (*P*<0.001 (***), and *P*<0.0001 (****)) calculated using either two-tailed Student's *t*-test (**e**,**f**), or one-way ANOVA (**a**,**d**) followed by Tukey's honest significant difference test. ANOVA, analysis of variance.

AUX/IAA transcriptional repressors exist as sister pairs, or ohnologues, with high sequence similarity, which have been retained in an unusually high proportion of cases after whole-genome duplication events, and have therefore been diverging for the same length of time^29^,^30^ (Supplementary Fig. 1). Functional shifts by neo- or sub-functionalization or selection for dosage balance in protein complexes contribute to the retention of such gene duplicates^31^. Among 29 AUX/IAA proteins in *Arabidopsis*, IAA6 and IAA19 ohnologues carry a degron motif and share high sequence identity (61.4%) (Supplementary Data 1). Nevertheless, dominant degron mutations, *iaa6/shy1* and *iaa19/msg2,* and swapping IAA6 and IAA19 N-terminal repressor domains (DI) indicate that IAA6 and IAA19 have distinct as well as shared functions in auxin signalling^17^,^32^,^33^,^34^,^35^,^36^. As *IAA6* and *IAA19* gene expression might reflect specific functions at the molecular level, we compared available data on mRNA expression profiles in different tissues, developmental stages, and *Arabidopsis thaliana* accessions (Fig. 1a and Supplementary Fig. 2a–c, Supplementary Note 1). Consistently, *IAA19* exhibited significantly higher expression than *IAA6*, indicating that albeit their relative conserved promoter regions^29^, the two genes are differentially regulated. Selective constraints on gene-coding sequences have been shown to increase with expression level^31^. As *IAA6* and *IAA19* orthologs are not present in *Carica papaya*, the duplication event seems to have occurred after *Brassicaceae* and *Caricaceae* separated. In the most simple scenario, one of the two sister genes keeps the function of the original single-copy gene in the last common ancestor, while the other gene either pseudogenizes or is free to sub- or neo-functionalize. Pseudogenization in this case has obviously not occurred. As *IAA19* expression is significantly higher than *IAA6*, *IAA19* is likely the gene that retained the original function. It is often possible to detect this trend by testing for positive selection between the two sister genes. However, the evolutionary signal present in these sequences among four *Brassicaceae* orthologues for each of the genes was not strong enough (or not present) to identify significant signatures of positive selection (based on the branch-site model in CodeML from the PAML package (version 4.9c))^37^. We therefore asked whether sequence divergence between the two genes differs by comparing the *IAA6* and *IAA19* orthologous *Brassicacae* sequences for each gene separately (Fig. 1b). While both full length sequences seemed rather conserved between the four *Brassicaceaes* tested (overall d*N*/d*S* *IAA6*=0.132; d*N*/d*S* *IAA19*=0.087), sliding window analyses revealed regions of increased sequence divergence in *IAA6*. These encompass the upstream region of the core degron and a conspicuous peak (d*N*/d*S*>100) in the PB1 domain (Fig. 1b). Since *IAA6* and *IAA19* orthologous sequences lacked indels in the vast majority of comparisons, these peaks must be driven by amino acid substitutions. A similar trend can be observed when intraspecific sequence divergence based on 80 resequenced *A. thaliana* accessions is assessed. Here, *IAA19* is once more highly conserved (d*N*/d*S*=0.169), while *IAA6* seems to be under relaxed selective constraints (d*N*/d*S*=0.660). Hence, although comparison of *IAA6* with *IAA19* did not reveal direct evidence for positive selection, *IAA6* but not the highly expressed *IAA19* includes regions with extensive sequence variation between *Brassicaceaes* when gene sequences were analysed separately (Fig. 1b and Supplementary Data 2). In addition, relaxed selective constraints indicate that within the *A. thaliana* germplasm *IAA6* may be in the process of sub-functionalizing.

### TIR1-IAA6 and TIR1-IAA19 receptors discriminate auxin levels

To address functional differences on the protein level, we then asked whether IAA6 and IAA19 vary in their potential to interact with TIR1, and AFBs in response to auxin in conventional yeast two-hybrid assays (Y2H). IAA6 and IAA19 interacted in an auxin-dependent manner with TIR1, AFB1 and AFB2 (Fig. 1c, Supplementary Figs 3 and 4). Particularly, low micromolar concentrations of naturally occurring auxins IAA, 4-chloro-IAA and, to a lesser extent, the synthetic auxin 1-naphthalene acetic acid (1-NAA) triggered TIR1/AFB1/AFB2-IAA6/19 associations (Fig. 1c and Supplementary Fig. 3). Overall, IAA19 interacted more strongly with TIR1/AFBs than IAA6, demonstrating that IAA6 and IAA19 differ in strength of auxin-dependent TIR1/AFB-AUX/IAA interactions. We hypothesize that these differences might arise from the unique amino acids in their degron-flanking regions (Supplementary Data 1), which may affect AUX/IAA ability to assemble into auxin co-receptor complexes.

Since it is possible that TIR1-IAA6 and TIR1-IAA19 co-receptors exhibit biochemical differences that enable specialized functions, we next assessed their auxin-binding properties via saturation binding assays using increasing concentrations of radiolabelled IAA (Fig. 1d,e). TIR1-IAA19 binds IAA with a *K*_d_ of ∼15.6 nM compared to a *K*_d_ ∼72.0 nM by TIR1-IAA6, indicating that TIR1-IAA19 co-receptor has a comparatively higher affinity for IAA than TIR1-IAA6 (Fig. 1e, Supplementary Figs 5 and 6). TIR1-auxin-AUX/IAA ternary complex formation was significantly compromised when the receptors consisted of TIR1-iaa19/msg2-1, or -iaa6/shy1-1 dominant mutants (Fig. 1c,d). We then directly compared the auxin affinity of TIR1-IAA6 and TIR1-IAA19 co-receptors via competitive binding assays, and determined *IC*_50_ and *K*_i_ values for each of the complexes using increasing concentrations of unlabeled IAA as competitor (Fig. 1f). At equilibrium, unlabeled IAA chased [^3^H]IAA consistently three times more efficiently from TIR1-IAA19 than TIR1-IAA6 (*K*_i_=33.5±3.7 nM and *K*_i_=99.3±11.9 nM, respectively), mirroring the affinity of the co-receptors for IAA determined in saturation binding experiments (Fig. 1f and Supplementary Fig. 7). Hence, IAA6 confers essentially lower auxin binding affinity than IAA19 to TIR1-AUX/IAA co-receptor complexes.

### Tracking specific SCF^TIR1^-mediated AUX/IAA ubiquitylation

**Figure 2:**
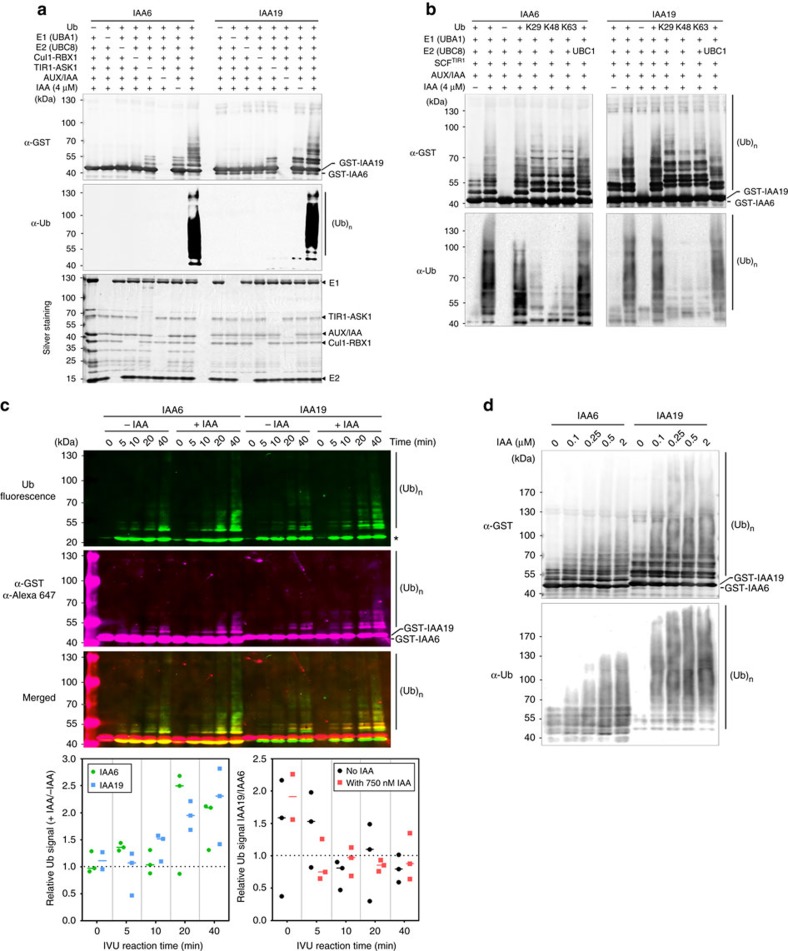
SCF^TIR1^-dependent and specific IAA6 and IAA19 ubiquitylation is enhanced by auxin. (**a**) IVU assays with recombinant GST-IAA6 or GST-IAA19, E1 (*At*UBA1), E2 (*At*UBC8), reconstituted SCF^TIR1^ (*At*SKP1-TIR1, *Hs*Cul1 and *Mm*RBX1), *At*Ub and IAA (auxin). AUX/IAA ubiquitylation is dependent on each component. Anti-GST, as well as anti-Ub antibodies recognize IAA6 and IAA19 ubiquitylated species. IVU reaction time 10 min (**b**) UBC8 and UBC1 Ub-conjugating enzymes (E2s) elicit poly-Ub-conjugation of IAA6 and IAA19 after 10 min *in vitro*. UBC8 promotes various Ub-linkages as seen by the reduction of IAA6 and IAA19 ubiquitylated species using either one of the chain-specific Ub-donors, Lys-29, Lys-48 or Lys-63 (Supplementary Fig. 9). (**c**) Rapid IVU of IAA6 and IAA19 is auxin- and time-dependent. Time course IVU reactions were performed using fluorescein isothiocyanate-labelled ubiquitin with or without 750 nM IAA. Ubiquitylation was monitored using the ubiquitin fluorescent signal (green, top) and Alexa Fluor 647-conjugated secondary antibodies for detection of GST:AUX/IAAs (magenta, middle). (*) Asterisk depicts ubiquitylated Cullin1 as previously reported^79^. ImageQuant TL software was used for quantification and generation of merged image (bottom). Ratios for auxin- and target-dependent ubiquitylation were calculated from three independent IVUs (*n*=3; see Supplementary Fig. 8b–d for replicates) and the single measurements are depicted in the corresponding scatter dot plots with line at median. A two-way ANOVA (*P*>0.05) showed no significant differences for the relative Ub signal between IAA6 and IAA19 in a specific time point (left), or for the IAA19/IAA6 ratio with or without 750 nM IAA. (**d**) Increased nanomolar concentrations of IAA promote IAA6 or IAA19 ubiquitylation after 10 min IVU reactions and higher molecular ubiquitylated species occurred on IAA19.

E3-target affinity determines a time interval in which Ub transfer to targets takes place^25^. Hereafter, we speculated that the strength of the SCF^TIR1^-IAA6 and SCF^TIR1^-IAA19 associations might impact AUX/IAA ubiquitylation and specifically, that the stability of SCF^TIR1^-AUX/IAA complexes affects the site of ubiquitylation, Ub-chain extension, or the dynamics of Ub-conjugation. To analyse Ub-conjugation dynamics, we developed a TIR1-dependent, cell-free *in vitro* ubiquitylation assay (IVU). A typical IVU consists of recombinantly expressed and highly purified E1 (*At*UBA1), E2 (mostly *At*UBC8), mammalian *Hs*Cul1-*Mm*RBX1 (ref. 38), Ub, *At*TIR1-ASK1 (ref. 9), and GST-tagged IAA6 or IAA19 targets (Fig. 2 and Supplementary Fig. 8). Thus, correct assembly of an *Hs*Cul1-*Mm*RBX1-ASK1-F-box^TIR1^ complex in our IVUs allows the recruitment and activation of a Ub-charged E2 (E2∼Ub) for Ub-conjugation of AUX/IAA *in vitro* (Supplementary Fig. 9).

To confirm the requirements for *in vitro* Ub-conjugation of IAA6 and IAA19, we pre-assembled SCF^TIR1^ complexes and performed IVUs when either one of the components was removed from the reaction. As expected, UBA1 (E1), UBC8 (E2), and SCF^TIR1^ (E3) were unambiguously required for IAA6 and IAA19 ubiquitylation (Fig. 2a). Moreover, SCF^TIR1^ showed strong E3 ligase activity *in vitro*. SCF^TIR1^ is a cullin-based RING ligase and since RING-E3s do not form a thioester intermediate with Ub, the linkage specificity of Ub-chain formation is likely conferred by the E2 (refs 39, 40). Therefore, the topology of Ub-chains assembled on a target by the RING-E3 can change with the nature of the E2 (refs 40, 41, 42). Also, while E1 function is universal and both *Arabidopsis* E1s (UBA1 and UBA2) show almost equal specificity in transferring activated Ub to a variety of *Arabidopsis* E2s (ref. 43), various E2-E3 combinations may affect E3 ligase activities. We then assessed how three E2s from different subclades out of the 37-member Ub E2 family in *Arabidopsis*^44^, namely UBC1, UBC4 and UBC8 catalyse Ub-conjugation to IAA6 and IAA19 (Fig. 2b and Supplementary Fig. 10). UBC1, 4 and 8 form a thioester linkage between the E2 and Ub, indicating these E2s can be charged with ubiquitin *in vitro* (Supplementary Fig. 9)^44^,^45^. Whereas, UBC1 and UBC8 triggered comparable IAA6 and IAA19 poly-ubiquitylation, only low molecular ubiquitin conjugates could be detected when using UBC4 as E2 in IVUs (Fig. 2b and Supplementary Fig. 10). This shows E2-SCF^TIR1^ selectivity and discrimination for auxin-mediated ubiquitylation of targets. These observations also suggest that the AUX/IAA ubiquitylation tracked in the IVU system is the consequence of the attachment of Ub polymers with different topologies. We therefore incorporated in our assays Ub variants bearing individually substituted lysine residues (K to R mutants), that have been widely used to characterize E2-E3 linkage specificity^46^. Hence, availability of a Ub mutant containing only a single lysine residue, either Lys29, Lys48 or Lys63 forces, if permitted by the E2-SCF^TIR1^ interaction, the formation of polyubiquitin chains on AUX/IAA targets via the single available lysine (Fig. 2b). We found that restricting ubiquitin concatenation leads to an alternate conjugation pattern, and there is an apparent loss of ubiquitin chain formation as compared with reactions containing wild-type ubiquitin (Fig. 2b). This implies ubiquitin conjugates on IAA6 and IAA19, in dependency of UBC8, are the product of different linkage types leading to alternative topologies, most likely several poly-mono-ubiquitylation and/or multi-, poly-ubiquitylation events. E2-E3 combinations determine specific chain formation by positioning the acceptor Ub in a defined orientation to favour linkage of the donor Ub on the selected lysine^25^. Therefore, it remains to be established, which E2-SCF^TIR1^ combinations occur, and whether Lys29, Lys48, Lys63 Ub-chains or a combination of them render IAA6 and IAA19 unstable *in vivo*.

### AUX/IAA ubiquitylation mirrors auxin receptor affinity

Next, we determined how IAA6 and IAA19 ubiquitylation is influenced by auxin. First, we monitored auxin-dependent ubiquitylation of AUX/IAAs over time using fluorescein-labelled ubiquitin, and fluorescent secondary antibodies for accurate and non-enzymatic detection of ubiquitin conjugates in a single image. We detected steady and rapid (<10 min) Ub-conjugation to IAA6 and IAA19 in the presence of auxin (750 nM IAA) (Fig. 2c and Supplementary Fig. 8b–d). Albeit much less efficient, as depicted by the relative Ub signal (+IAA/−IAA) (depicted in lower panel Fig. 2c), we observed AUX/IAA ubiquitylation in the absence of IAA, which is probably the result of basal interactions between SCF^TIR1^ and AUX/IAAs^7^,^8^,^9^,^11^,^47^,^48^,^49^. IVU reactions in the presence of ∼10 × and ∼50 × [IAA] higher than the observed auxin affinity of TIR1-IAA6 and TIR1-IAA19 co-receptor complexes, respectively (Fig. 1e), did not provide evidence for significant differences in the ubiquitylation status of IAA19 over IAA6 (depicted in lower panel Fig. 2c and Supplementary Fig. 8b–d). Intriguingly, when we further evaluated ubiquitylation of AUX/IAAs with increasing nanomolar concentrations of IAA, we detected a surge in high molecular weight species in IAA19 compared to IAA6 (Fig. 2d). While a steady increase in Ub-conjugation of IAA6 took place at 0.1–2 μM [IAA] after 10 min, Ub-conjugation of IAA19 spiked already at the lowest IAA concentration (Fig. 2d and Supplementary Fig. 8e,f). This suggests a greater efficiency of the ubiquitylation machinery acting upon IAA19 at low auxin concentrations. Taken together, these experiments are the first to demonstrate reconstitution of SCF^TIR1^ assembly and AUX/IAA ubiquitylation.

### AUX/IAA Ub-site selection depends on local flexibility

**Figure 3:**
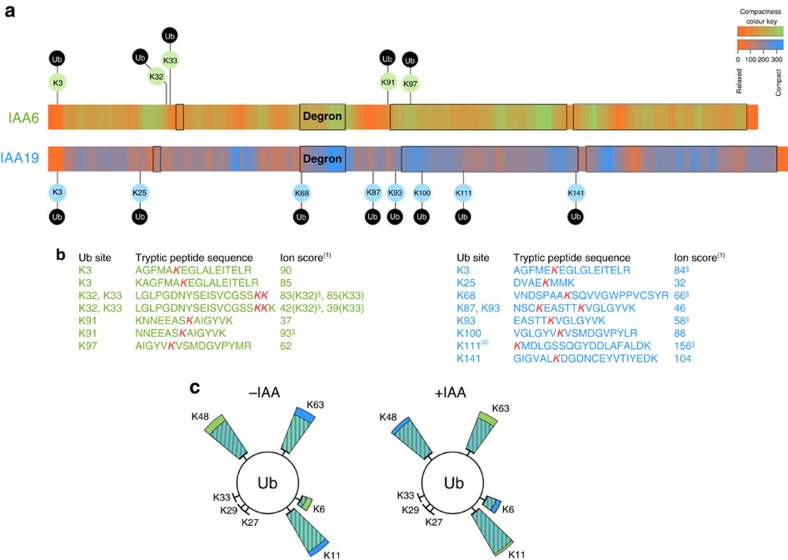
IAA6 and IAA19 exhibit high intrinsic disorder offering a broad ubiquitylation zone with likely limited lysine availability. (**a**) Putative lysine ubiquitylation in IAA6 and IAA19 concentrate in hotspots with low compactness. Meta-structure of IAA6 and IAA19 was quantified by sequence-derived parameters, compactness and local secondary structure. Residue-specific compactness is displayed in green-orange (IAA6), and blue-orange (IAA19) 2-colour sequential variation (see colour key), where folding corresponds to values above 300 from DisProt database (see Supplementary Fig. 14 for details). IAA6 and IAA19 IVU samples were analysed via LC-MS and putative ubiquitylation sites were mapped relative to their domain structure (black boxes). (**b**) List of Ub-modified IAA6 (green) and IAA19 (blue) peptides and their ion scores (Mascot^1^) identified by mass spectrometry. Specific ubiquitylated Lys-residues of cleaved peptides are shown in red, and (§) symbol depicts the site is also covered by the Lys-Arg-Gly-Gly (LRGG) remnant, which is further confirmation that the site is genuine. Ub-conjugation on Lys111 in IAA19 (ref. 2) is also supported by the LRGG remnant, reducing the uncertainty caused by the N-terminal location on the peptide. See Supplementary Figs. 11 and 12 for information about reproducibility and FDR. (**c**) Distribution of identified ubiquitin linkage types. IVU reactions for IAA6 (green) and IAA19 (blue) with or without IAA were analysed via LC-MS, and ubiquitin peptides corresponding to different ubiquitin linkage types were identified (for details see Supplementary Fig. 13).

Having developed a tool for investigating IAA6 and IAA19 recognition by the SCF^TIR1^-E3 ligase and subsequent ubiquitylation *in vitro*, we next sought to determine the residue(s) within IAA6 and IAA19 that function as attachment sites for Ub (Fig. 3a,b). We processed IVU samples containing IAA6- and IAA19-ubiquitin conjugates for liquid chromatography coupled with tandem mass spectrometry (LC-MS) and inspected MS/MS spectra for peaks with a mass difference representing LRGG (trypsin miscleavage product of Ub C-terminus) and di-Gly modified residues (Fig. 3b, Supplementary Figs 11–13 and Supplementary Table 1, Supplementary Table 2). We found Lys-ubiquitylation on IAA6 and IAA19 in regions with low or intermediate compactness (Fig. 3a and Supplementary Fig. 14a,b) and more Ub-modified peptides for IAA19 than for IAA6 independently of auxin present in the IVU reactions. Reproducible ubiquitylated sites in independent replicates comprise Lys3, Lys32, Lys33, Lys91 and Lys97 in IAA6 (27% total Lys); and Lys3, Lys25, Lys68, Lys87, Lys93, Lys100, Lys111 and Lys141 in IAA19 (47% total Lys) (Fig. 3a,b). Ubiquitylated Lys3 is a conserved residue among a subgroup of AUX/IAAs including IAA6, 19, 8, 9, 34, 32. Neighbouring Lys32 and Lys33 in IAA6 appear to be equivalent to Lys25 in IAA19. These residues are located in the vicinity of the completely conserved but not ubiquitylated KR motif in a region decorated with additional multiple unmodified lysines (Supplementary Data 1). Similarly, ubiquitylated Lys91 in IAA6 coincides with K87 and K93 in IAA19, also located in a region downstream of the canonical degron including a rate motif and DIII in the PB1 domain. Specifically, ubiquitin modified Lys97 in IAA6 akin Lys100 in IAA19 are completely conserved among *Arabidopsis* AUX/IAAs, which encourages the idea that this is a common ubiquitylation site in the AUX/IAA family (Supplementary Data 1). Interestingly, ubiquitylation of Lys97 of IAA6, and Lys100 and Lys111 of IAA19 could serve as a mechanism to dislodge AUX/IAA interaction partners by interfering with their oligomerization interface (Supplementary Fig. 14c,d). In addition, although these are highly or completely conserved residues, we did not identify Lys68, or Lys111 ubiquitylated peptides in IAA6. We therefore cannot rule out that our MS-based analysis might be affected by the fidelity of the ubiquitylation *in vitro*, permitting only a subset of possible ubiquitylation sites to be detected. Non-canonical ubiquitylation of AUX/IAAs was previously proposed, as substitution of 16 lysines in IAA1 is not sufficient to abrogate its localization, turnover and function^50^. In our assays non-canonical IAA6 and IAA19 ubiquitylation might not be favoured, due to its low probability, the relative instability of the thioester bond to Cys in MS analysis, and the less frequent and also less kinetically stable hydroxyester linkages to Ser, and Thr^51^. Nevertheless, IAA6 and IAA19 ubiquitylation might rather depend on the structural adaptability around the ubiquitylation surface, namely local flexibility, enabling a choice of multiple lysines to be modified^27^,^52^. Concertedly, the *in vitro* tracking of Lys-ubiquitylation on IAA6 and IAA19 is placed on putatively exposed flexible regions flanking structured domains, so that AUX/IAA Ub-site selection depends on a specific local environment (Fig. 3a and Supplementary Fig. 14). Thus, our data nicely support recent findings showing that Ub-sites on targets exhibit striking propensity to occur within intrinsically disordered regions in a specific determinant sequence neighborhood^27^.

Various linkages of polyubiquitin chains which are determined either by the E2 or less frequently, by the E3 ligase^53^, confer distinct fates to target proteins^54^. Therefore, we surveyed the relative abundance of ubiquitin linkage types in our IVUs by making a direct estimate from the number and frequency of peptide spectrum matches (PSMs) from ubiquitylated lysine residues in ubiquitin. Independently of auxin, primarily K48-, K11-, K63-, and to a much lesser extent K6-linked chains were identified in the samples (Fig. 3c and Supplementary Fig. 13). It has been shown that ubiquitin chains on targets adopt either compact or open conformations affecting the proteasome ability to unfold and degrade the target^55^. So, K48- or mixed linkage-chains, adopting compact conformations, lead to a greater turnover than K63-linked chains^54^. Combinations of homologous, heterologous and branched ubiquitin chains on IAA6 and IAA19 possibly endow their degradation by the proteasome.

### Auxin co-receptor affinity tunes AUX/IAA turnover

**Figure 4:**
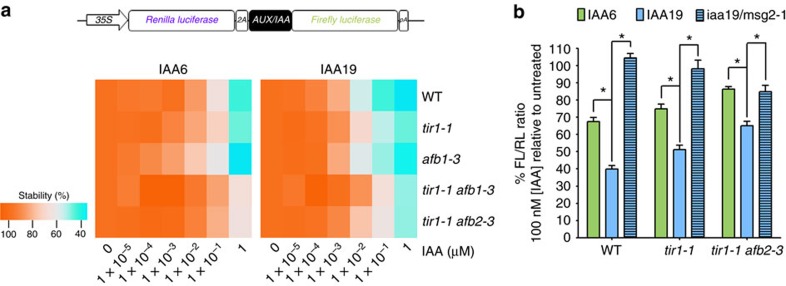
IAA6 and IAA19 stability is differentially impacted by their ability to form auxin coreceptor complexes with variations in affinity. (**a**) Ratiometric luminescence auxin biosensor constructs comprising IAA6 or IAA19 coding sequences flanked by Renilla luciferase (RL), and a C-terminal fusion with Firefly luciferase (FL) under the 35S constitutive promoter. 2A oligopeptide (2A) and poly(A) tail (pA) elements allow stoichiometric co-expression of RL and IAA6 or IAA19 FL fusions, and maturation of messenger RNA for their translation, respectively. *A. thaliana* protoplasts of *Col-0* (wild type, WT), *tir1-1, afb1-3* single and *tir1-1 afb2-3* and *tir1-1 afb3-4* double mutant plants transformed with IAA6 or IAA19 auxin biosensors were incubated for 30 min in IAA-supplemented medium (10 pM–1 μM IAA) prior luciferase activity determination. Auxin dose response on AUX/IAA stability is calculated as percentage of a decrease in FL/RL of untreated samples at a given IAA concentration. Heat map displays means (*n*=6) of FL/RL ratios of IAA6 and IAA19 sensors. Detailed graphs for each sensor in each genotype are shown in Supplementary Fig. 15. (**b**) Sensitivity of IAA6, IAA19 or IAA19^P76S/msg2-1^ sensors in protoplasts of *Col-0* (WT), *tir1-1*, and *tir1-1 afb2-3* plants. Ratiometric luciferase activities are shown as percentage (%) of FL/RL ratio at 100 nM [IAA] relative to untreated samples. Statistical differences (*) were calculated by two-way ANOVA of the absolute data. Error bars, s.e.m. Data were considered statistically significant if the *P* value was <0.05.

*In vivo,* many factors may influence auxin co-receptor formation and IAA6 and IAA19 processing. Therefore, we quantitatively assessed IAA6 and IAA19 degradation in various TIR1/AFB mutant backgrounds, and monitored their response to auxin. We generated IAA6 and IAA19 ratiometric luminescent sensor constructs^56^ for transient expression in *Arabidopsis* leaf protoplasts, and measured auxin-dependent degradation as a decrease in firefly relative to renilla luminescence (FL/RL ratio) (Fig. 4 and Supplementary Figs 15 and 16). IAA6 and IAA19 sensors showed auxin concentration-dependent degradation in the wild-type genetic background, rapidly responding towards low levels of exogenously applied IAA. While IAA concentrations between 100 pM to 1 nM triggered IAA19 degradation, 10 nM IAA was required for comparable turnover of IAA6 (Supplementary Fig. 15). In *tir1-1* and *tir1-1 afb2-3* or *tir1-1 afb3-4* double mutant backgrounds, IAA6 and IAA19 degradation was reduced, requiring ∼10 times more IAA to reach wild-type degradation rates (Fig. 4a and Supplementary Fig. 15). Interestingly, the differences we observed between IAA6 and IAA19 coincide with estimates for relative speed of auxin-induced turnover for IAA6 and IAA19 in a synthetic approach^57^. Additionally, incorporating MG132 proteasome inhibitor stabilized IAA6 and IAA19 (Supplementary Fig. 16b). Thus, degradation of IAA6 and IAA19 sensors in our protoplast system is proteasome-dependent consistent with previous observations^58^, and sensors carrying dominant mutations in the degron displayed increased stability (Supplementary Fig. 16a). Also, specific structural features of IAA6 and IAA19 might contribute to fine-tuning their turnover. A structural approach in the future will surely corroborate whether rate motifs^18^ on IAA6 and IAA19 degron-flanking regions amplify or mitigate turnover dynamics. For instance, slightly longer rate motifs enriched with Gly residues in IAA19 (Supplementary Fig. 1b) could eventually confer much more flexibility, so that amino acid composition affects the conformational ensemble and facilitates processivity on IAA19.

## Discussion

**Figure 5:**
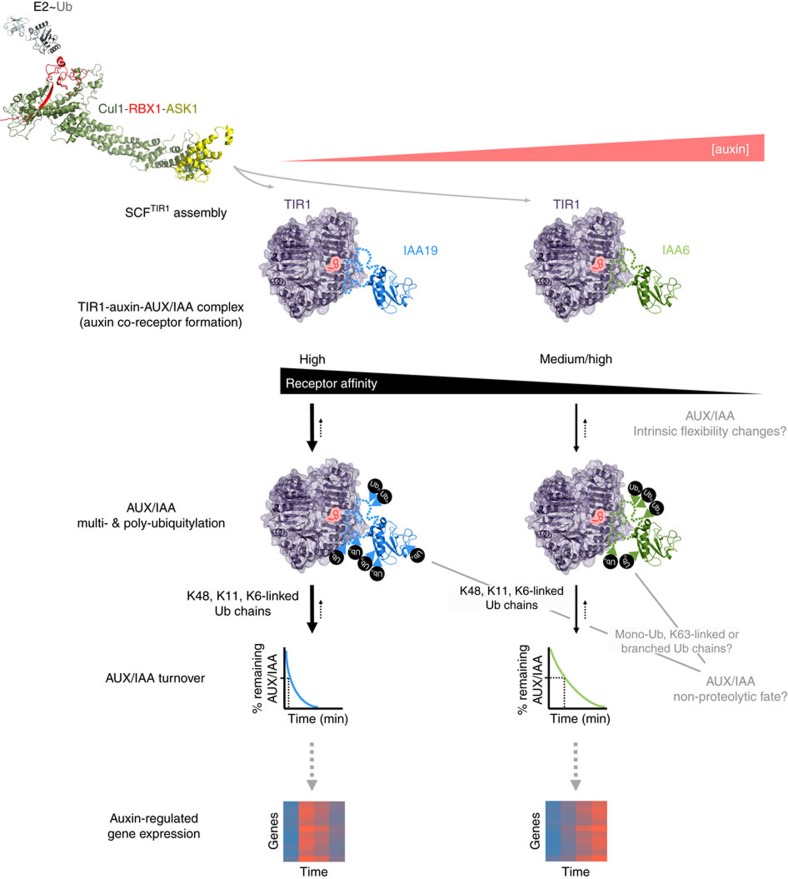
Simplified model of auxin sensing by SCF^TIR1^-IAA6 and SCF^TIR1^-IAA19 co-receptor complexes. A Cullin-RING E3 ligase^80^ from the SCF-type is formed when TIR1 or its paralogs AFB1-5 interchangeably assemble with the adaptor protein ASK1. SCF^TIR1^ interacts with both E2∼Ub and IAA6 or IAA19 degradation targets in response to intracellular auxin levels. TIR1 recruits IAA19 at low nanomolar concentrations of IAA and forms a high affinity co-receptor complex, while TIR1-IAA6 displays only a medium IAA affinity. Co-receptor complex dissociation is possible but unfavored in the presence of auxin (reverse dotted arrows). A degron and intrinsically disordered regions (unstructured dotted line) most likely fit on top of auxin in the TIR1-auxin-binding groove. It is currently unknown whether the two-pronged PB1-like IAA6 and IAA19 homo- and heterodimerization domains III-IV (folded structure) directly contribute to auxin binding. IAA binding affinities of TIR1-IAA6 and TIR1-IAA19 complexes yield differential Ub-conjugation at different sites. Lysines (K) along the IAA19 structure probably become ubiquitylated with Lys48-, Lys6-, Lys11-chain linkage types offering multiple ubiquitylation signatures for efficient and rapid degradation by the 26S proteasome. Putatively IAA6 ubiquitylation on lysine residues might be less efficient, leading to a comparably slower IAA6 turnover. Other residues in flexible and/or intrinsically disordered regions of IAA6 and IAA19 eventually become ubiquitylated *in vivo*. Since the outcome of AUX/IAA ubiquitylation depends on the distinct types of ubiquitin topologies, K63-linked ubiquitin chains, monoubiquitylation or mixed chains on IAA6 and IAA19 could affect their function and have a non-proteolytic role. Conceivably, AUX/IAA ubiquitylation can be counteracted by the activity of deubiquitylases (reverse dotted arrows). AUX/IAA ubiquitylation, particularly initial rounds, might trigger temporal- and auxin- dependent SCF^TIR1^-AUX/IAA binding specificity variations through intrinsic flexibility changes. IAA19 has a very short half-life, its ohnologue IAA6, although also unstable, exhibits longer half-life, which is a reflection of their differential affinity for auxin when in TIR1-containing co-receptor complexes. Consequently, IAA6- and IAA19-dependent specific transcriptional outputs, in different tissues and in response to different auxin concentrations, are likely impacted by AUX/IAA processing.

Here, we propose a model (Fig. 5) in which IAA6 and IAA19 ohnologues have evolved functionally specialized auxin sensitivity through differential auxin co-receptor formation, auxin sensing, and ubiquitylation. Despite high amino acid sequence similarity, IAA19 associates more strongly with TIR1/AFBs than IAA6 does, forms a higher affinity TIR1-auxin-IAA19 ternary complex, and is ubiquitylated with higher processivity at lower auxin concentrations. As ubiquitylation is highly dynamic, SCF^TIR1^ complex formation and stability as well as AUX/IAA isomerization and deubiquitylation may also affect IAA6 and IAA19 Ub-conjugation status, pacing their processing and degradation dynamics in a cellular context.

Our studies on the dynamics of TIR1-IAA6 and TIR1-IAA19 co-receptor formation and outcome suggest that a subtle AUX/IAA sequence divergence drives functional specialization, thereby dictating AUX/IAA Ub-conjugation, and most likely degradation. Thus, these events ultimately impinge on ARF interactions and auxin-dependent gene activation. It is quite remarkable that differences between sister genes like *IAA6* and *IAA19* might already leave traces on both expression level, and sequence divergence of each single gene. Regions of increased sequence divergence in *IAA6* coincide with ubiquitylation hotspots in *IAA19*. Whether these regions in *IAA6* with relaxed selection have a functional relevance and provide, for instance, a different landscape for ubiquitin conjugation affecting AUX/IAA stability, or are merely an effect of genetic drift remains, so far unknown.

The higher ubiquitylation processivity we observed for IAA19 compared to IAA6 in response to auxin may be a function of higher auxin affinity of TIR1-IAA9 versus TIR1-IAA6. Higher auxin affinity likely confers greater stability to the SCF^TIR1^-IAA19 interaction, which may prolong the time interval in which IAA19 is available to the E3 ligase for Ub-conjugation. Structural constraints may preclude targeting residues limiting the E3's area of action^25^, so alternative and differential IAA6 and IAA19 ubiquitylation could depend on how such residues are available in IAA6 and IAA19 ubiquitylation zones^59^,^60^. Interestingly, some E3s generate ubiquitin-rich foci on proteins that act as stable recruitment platforms for DNA and/or cognate protein partners^55^. For instance, multi-monoubiquitylation or Lys63-linked chains act as transient mediators of protein interactions^61^. The relevance of such Ub-modifications on IAA6 and IAA19 remains to be determined in future studies. Our results allow us to postulate that the UBC8-SCF^TIR1^ combination yields Ub-chains on IAA6 and IAA19 that most presumably confer recognition by the proteasome and a degradation outcome.

We propose that although a single polyubiquitin chain on one Ub-site might be sufficient for targeting IAA6 and IAA19 for degradation, the relative location of additional ubiquitylation sites such as Lys particularly in flexible regions serve as backup sites for differential ubiquitylation in response to auxin. We demonstrate that SCF^TIR1^-mediated ubiquitylation of IAA6 and IAA19 can occur via lysine residues on flexible disordered regions, each of which could be sufficient to induce the rapid degradation of IAA6 and IAA19 *in vivo*. Given the vast scope for variation in Ub-linkage types and their associated topologies, it is also plausible that only specifically linked Ub-chains on IAA6 and IAA19 via isopeptide bonds at certain lysines result in proteasomal degradation. Conversely, mono-, multi-monoubiquitylation or poly-ubiquitylation with distinct Ub-chain topology might alter AUX/IAA localization, and/or its intrinsic properties thereby conditioning IAA6 and IAA19 turnover in a cellular environment. Alternatively, the same events leading to differential AUX/IAA ubiquitylation might regulate auxin signalling non-proteolytically by controlling AUX/IAA activity or offering a signal for recruiting or modulating interaction with partners such as ARFs.

Together, we combined quantitative *in vitro* and *in vivo* tools to reveal underlying mechanisms and consequences of discriminatory auxin perception and response. In the future, combining genetic studies of early-diverging land plants with biochemical tools, such as those we have developed and implemented here, will surely give a unique insight into the evolution, dynamics and the wiring of the auxin response system. Our results illustrate how evolution of primary protein structure may be amplified through interaction with small molecules and protein complexes downstream. In our system, the consequence of these differential interactions is distinct degradation kinetics of transcriptional repressors central to auxin response. It is likely that similar mechanisms specify responses among not only the other AUX/IAA proteins, but also among the many other protein families that participate in small molecule sensing. Thus, we offer a model strategy for interpretation of small molecule concentrations into fine-tuned control of gene expression.

## Methods

### Population genetic and gene expression analyses

AtGenExpress (http://jsp.weigelworld.org/AtGenExpress/resources/) and *Arabidopsis* expression data deposited at the eFP browser (http://www.bar.utoronto.ca/) were used to retrieve and compare *A. thaliana* expression profiles for *IAA6* and *IAA19* in different tissues (full citation list in Supplementary Note 1), developmental stages^62^ and natural accessions^63^.

### Sequence divergence between *Brassicaceaes*

*IAA6* and *IAA19 A. thaliana* sequences and the BLASTp (BLAST version 2.2.21) reciprocal best hit in *A. lyrata, A. halleri and C. rubella* were used to generate sequence alignments using the L-INS-i option in MAFFT^64^. The resulting protein alignment and the corresponding nucleotide sequences were used to compute codon alignments with Pal2Nal (ref. 65). Based on the codon alignments, nucleotide divergence was computed with a sliding window analysis (window size: 50, step: 3) with DnaSPv5.1 (ref. 66).

### Phylogeny of AUX/IAA proteins in *A. thaliana*

*A. thaliana* AUX/IAA amino acid sequences were aligned using the L-INS-i option in MAFFT^64^. JTT+F+G was selected as best fitting amino acid substitution model according to the Bayesian Information Criterion in the MEGA-CC Model Selection analysis^67^. To reconstruct the phylogeny, the maximum likelihood (ML) algorithm with a bootstrap test (1000 replications) implemented in MEGA-CC was applied (additional settings: No of Discrete Gamma Categories=5, Site Coverage Cutoff (%)=95, ML Heuristic Method=Nearest-Neighbor-Interchange (NNI), Initial Tree for ML=Make initial tree automatically, Branch Swap Filter=None, Gaps/Missing Data Treatment=Partial deletion). The unrooted phylogenetic tree was obtained with MEGA Tree Explorer^68^.

### Protein expression and purification

Preparations of recombinantly expressed GST-tagged ASK1-TIR1 protein complex from SF9 insect cells were essentially performed as previously published^9^,^11^. GST-tagged *Arabidopsis* AUX/IAAs were expressed in *Escherichia coli* BL21 (DE3) cells carrying N-terminal GST-tagged IAA6 (AT1G52830) and IAA19 (AT3G15540) plasmids. Cells were harvested by centrifugation (5000g, 15 min) and resuspended in lysis buffer (50 mM Tris pH 8.0, 200 mM NaCl, 2.5 mM DTT, and cOmplete EDTA-free protease inhibitor (Roche)). After lysis by sonication, lysates were cleared by centrifugation, and the supernatant was used for purification either via gravity flow using GSH agarose (SERVA), or via an ÄKTA pure FPLC system using a GSTrap 4B column 1 ml (GE Healthcare). The supernatants were loaded on the column, washed with at least 5 column volumes (CV) of lysis buffer. GST-tagged proteins were eluted using 10 mM glutathione in lysis buffer, and corresponding fractions were pooled, concentrated, buffer exchanged to lysis buffer containing 15% glycerol and stored at 4 °C until use or directly used.

GST-tagged ASK1-TIR1 was expressed in Sf9 or Hi5 insect cells and purified in a similar fashion. After affinity purification using a FPLC system, the GST-tag was cleaved of by TEV protease treatment and further purified using anion exchange (MonoQ, GE Healthcare) and gel filtration chromatography (Superdex 200, GE Healthcare). Appropriate fractions were pooled, buffer exchanged to glycerol-containing buffer, concentrated, frozen in liquid nitrogen and stored at −80 °C until use.

6xHis-UBA1 and 6xHis-UBC8 were expressed and purified from *E. coli* BL21-AI after 5 h of induction (0.01% L-Arabinose) at 28 and 22 °C, respectively. Cells were lysed in 25 mM Tris-HCl, pH 8.0, 500 mM NaCl, 20 mM imidazole, 2 mM DTT, 1 mM EDTA, 1 mM PMSF, protease inhibitor cocktail (Roche, cOmplete mini, EDTA-free). Cleared lysates were supplemented with 5 mM MgCl_2_ and loaded onto a pre-equilibrated (wash buffer: 25 mM Tris-HCl, pH 8.0, 350 mM NaCl, 20 mM imidazole, 2 mM DTT) HisTrap FF 5 ml column (GE Healthcare) at 2 ml min^−1^. The column was washed with 5 CV of wash buffer including 65 and 100 mM imidazole for 6xHis-UBA1 and 6xHis-UBC8, respectively. 6xHis-UBC8 was eluted with 25 mM Tris-HCl, pH 8.0, 350 mM NaCl, 400 mM imidazole, 2 mM DTT, whereas 6xHis-UBA1 was eluted with 250 mM imidazole in the same buffer. 6xHis-UBC8 was concentrated by centrifugation (10 kDa MWCO Centricon, Millipore), dialyzed and finally stored in 50 mM Tris-HCl, pH 8.0, 200 mM NaCl, 2 mM DTT and 25% (v/v) GlyOH. Elution fractions of 6xHis-UBA1 were combined, diluted with 15 volumes of anIEX equilibration buffer (50 mM Tris-HCl, pH 8.0, 5 mM NaCl, 2 mM DTT) and applied to a HiTrap Q XL 1 ml column (GE Healthcare). Elution was initiated without any wash step by a linear gradient from 5 mM NaCl to 1 M NaCl (0–100% anIEX elution buffer in 50 CV; 50 mM Tris-HCl, pH 8.0, 1 M NaCl, 2 mM DTT). 6xHis-UBA1 eluted at a salt concentration of ∼330 mM NaCl. Appropriate fractions were pooled, concentrated and loaded onto a HiLoad S200 16/60 pg (GE Healthcare). 6xHis-UBA1 eluted at a retention volume of ∼65 ml. 6xHis-UBA1-containing fractions were pooled, concentrated and stored as described for 6xHis-UBC8.

*Hs*Cul1-*Mm*RBX1 purification was performed using the split'n coexpress system^69^. Briefly, *E. coli* BL21 (DE3) cells expressing GST-tagged *Hs*Cul1-*Mm*RBX1 were harvested, resuspended (for buffer composition, see GST-AUX/IAA purification) and lysed by sonication. Cleared lysate was subjected to affinity chromatography using a GSTrap 4B 5 ml (ÄKTA system, GE Healthcare). Appropriate fractions were pooled, concentrated and incubated with thrombin (SERVA, see manufacturer's protocol for cleavage conditions). After dilution to approx. 40 mM NaCl, the solution was subjected to anion exchange and gel filtration chromatography. *Hs*Cul1-*Mm*RBX1-containing fractions were pooled, buffer exchanged to 15% glycerol-containing lysis buffer, frozen in liquid nitrogen and stored at −80 °C.

### [^3^H]-labelled auxin binding assay

Radioligand binding assays were performed using 20 nM purified ASK1-TIR1 protein complexes, as well as 2–15 μM GST-tagged AUX/IAAs (except Supplementary Fig. 88e,f, where GST has been cleaved off IAA6) or their GST-aux/iaa dominant mutant versions, and [^3^H]IAA with a specific activity of 25 Ci/mmol from American Radiolabeled Chemicals, Inc. All reactions were carried out in a volume of 100 μl (for additional details see^11^,^70^). Nonspecific binding was determined using at least 500 × excess of cold IAA with respect to [^3^H]IAA. Specific binding was calculated as the average of at least two measurements of nonspecific binding subtracted from total binding. For saturation-binding assays, samples were prepared as above and incubated with at least six IAA concentrations on either side of the *K*_d_ of a given co-receptor pair. The saturation-binding curves were fitted to the Morrison equation for tight binding^71^. Since nonspecific binding exceeded 10% of total binding in all independent experiments, total binding data were additionally analysed according to Swillens, *Mol Pharm*, 1995 (ref. 72). For homologous competition binding assays, ASK1-TIR1 as well as GST-tagged AUX/IAA proteins were incubated with a fix concentration of either 50 or 25 nM [^3^H]IAA for experiments with IAA6 and IAA19, respectively. Data of three independent experiments (*n*=3) were plotted against the concentration of cold IAA and fitted with built-in analysis (one-site fit logIC50) of Prism5, GraphPad Software, Inc. Importantly, formation of ASK1-TIR1-IAA-AUX/IAA complexes cannot be strictly described using the above models *per se*. Auxin co-receptor complex formation is expected to be consisting of reversible binding events with yet unknown hierarchy. An intuitive model would assume that TIR1 and auxin form first a TIR1:auxin complex. This partial reaction is described by the dissociation constant *K*_*D*_^*auxin*^. Next, the TIR1:auxin complex binds the AUX/IAA with a high-affinity *K*_*D*_^*AUX/IAA*^. Using an excess of AUX/IAA over TIR1, thus allows to assume a bimolecular association between ASK1-TIR1-AUX/IAA co-receptors and [^3^H]IAA. In radioligand binding assays, neither dissociation constant of the partial reactions is assessable. Therefore, it can be assumed that in the auxin binding assays, one actually determines the apparent dissociation constant *K*_*D*_' for ternary complex formation from the reactants, *i.e.* the net binding reaction.

### E2-charging assays

Reactions for E2∼Ub thioester formation were performed using 50 μM *At*Ubiquitin (*At*Ub) or *Hs*Ubiquitin mutants containing one Lys residue available (Boston Biochem, UM-HK480-01M, UM-HK630-01M, UM-HK290-01M), 2 μM of 6 × His-UBA1 and 20 μM 6xHis-E2 protein (UBC1, UBC4 or UBC8) mixed in thioester buffer (10 mM HEPES, pH 7.5, 100 mM NaCl, 4 mM ATP and 20 mM MgCl_2_). Reactions were incubated at room temperature for 10 min, and subsequently mixed with reducing (containing 40 mM DTT) or non-reducing (without DTT) SDS-sample buffer. Samples were boiled for another 10 min and afterwards resolved by 15% SDS-PAGE followed by Coomassie staining or immunoblot detection using 1:500 or 1:1,000 dilution of monoclonal anti-Ub (P4D1) (Santa Cruz, SC-8017), and 1:10,000 anti-mouse HRP (Thermo Scientific, Cat. # 31430).

### *In vitro* reconstitution of Ub-conjugation

Proteins were prepared as described above and amounts are expressed relative to AUX/IAA concentrations ([AUX/IAA]). Two mixtures (mix A and mix B) were prepared in parallel. Mix A contained 7.5 to 10 fold molar excess of Ub (either 6xHis-*Hs*Ub or *At*Ub), 6xHis-*At*UBC8 (1x [AUX/IAA]) and 6xHis-Uba1 (0.1–0.2x [AUX/IAA]) in reaction buffer (30 mM Tris-HCl, pH 8.0, 100 mM NaCl, 2 mM DTT, 5 mM MgCl_2_, 1 μM ZnCl_2_, 2 mM ATP. Mix B was prepared by mixing 0.1x [AUX/IAA] of Cul1-RBX1, 0.1x [AUX/IAA] of ASK1-TIR1 with AUX/IAA in reaction buffer. Mix B was aliquoted and supplemented with indicated amounts of IAA. Mixtures A and B were separately incubated for 5 min at 25 °C with shaking at 500 rpm. Equal volumes of mix A and B were combined to initiate the ubiquitylation reaction (0 min). Aliquots were taken at specified time points and reactions were stopped by denaturation in 2X Laemmli buffer. Protein samples were electrophoretically separated in either 8% or 5–15% mini- or maxi- polyacrylamide gradient gels, and transferred onto nitrocellulose membranes. Immobilized Ub-conjugated proteins were detected with monoclonal anti-Ub (P4D1) as described above, or with 1:10,000 dilution of polyclonal anti-GST in rabbit (Sigma, G7781), and 1:10,000 anti-rabbit HRP in goat (Santa Cruz, SC-2004) as secondary antibody.

For quantification of ubiquitin conjugates, IVU reactions were performed as described above with the following modifications. 50 μM fluorescein-labelled ubiquitin (UBPBio, S20C) instead of *At*Ub was included in the reactions. IVU reactants were adjusted accordingly: Mix A contained 10 fold molar excess of fluorescein-labelled Ub, 6 × His-AtUBC8 (0.4x [AUX/IAA]) and 6xHis-Uba1 (0.04x [AUX/IAA]); and Mix B contained 0.2 × [AUX/IAA] of Cul1-RBX1, 0.2 × [AUX/IAA] of ASK1-TIR1 with AUX/IAA and 750 nM IAA. Mix A and Mix B were prepared in reaction buffer and IVU reactions were incubated at 25 °C between 0 and 40 min. IVU reactions were separated by SDS−polyacrylamideage followed by immunoblotting using primary anti-GST antibody (1:10,000; Sigma, G7781) and secondary anti-rabbit Alexa Fluor Plus 647 antibody (1:20,000) (Thermo Fischer Scientific, A32733). Nitrocellulose membranes were scanned using a Typhoon FLA9500 system (473 nm excitation wavelength and LPB filter for ubiquitin signal detection and 635 nm excitation wavelength and LPR filter for GST signal). Fluorescent signals located between GST-tagged AUX/IAAs, and ubiquitylated Cullin (∼50 kDa), which correspond to IAA6- and IAA19-ubiquitin conjugates (see Fig. 2c) were quantified for each lane using ImageQuant TL software automatic lane detection. Background subtracted signals were used to generate ratios between auxin-dependent and independent ubiquitylation of GST-IAA6 and GST-IAA19. In the same way, the relative ubiquitin signal corresponding to the ratios between ubiquitin conjugates on GST-IAA19 over GST-IAA6 were generated. Two fluorescence signals were excluded (T0) due to their low intensity, which otherwise would have resulted in artificial high ratios. To evaluate for significance, a two-way ANOVA with Bonferroni multiple comparisons post-tests was performed using GraphPad Prism software.

### LC-MS analyses

IVU reactions were performed as described above. Three sets of IVUs, corresponding to three independent (biological) replicates, were carried out on consecutive weeks using AUX/IAA proteins from different batch preparations. After 30 min, 20 μl IVUs were stopped by denaturing with 1 volume 16M urea. Proteins were further reduced by adding 0.5 μl of 200 mM dithiothreitol (DTT), and alkylated by adding 2 μl of 200 mM iodoacetamide. The reactions were quenched with 2 μl of 200 mM DTT, and subsequently 320 μl of 50 mM ammonium bicarbonate pH 8.5 were added. Alternatively, samples were also processed without reduction and alkylation. Proteins in the IVU reactions were digested with trypsin (enzyme to substrate 1:50 (w/w)) at 37 °C with gentle agitation overnight. Reactions were quenched by adding formic acid (FA) to a final concentration of 0.1%, and the peptides were desalted as previously described^73^. Dried peptides were dissolved in 5% acetonitrile, 0.1% trifluoric acid, and 0.5 μg were injected into the LC-MS system. Peptides were separated using liquid chromatography C18 reverse phase chemistry employing a 120 min gradient increasing from 5 to 40% acetonitrile in 0.1% FA, and a flow rate of 250 nl min^−1^. Eluted peptides were electrosprayed online into a QExactive Plus mass spectrometer (Thermo Fisher Scientific). The spray voltage was 1.9 kV, the capillary temperature 275 °C and the Z-Lens voltage 240 V. A full MS survey scan was carried out with chromatographic peak width set to 15 s, resolution 70,000, automatic gain control (AGC) 3E+06 and a max injection time (IT) of 200 ms. MS/MS peptide sequencing was performed using a Top10 DDA scan strategy with HCD fragmentation. MS/MS scans were acquired with resolution 17,500, AGC 5E+04, IT 150 ms, isolation width 1.6 *m/z*, normalized collision energy 28, under fill ratio 3%, dynamic exclusion duration 40 s, and an intensity threshold of 1E+04. Peptides were identified and ubiquitylated residues on identified peptides were mapped using both the Mascot software v2.5.0 (Matrix Science) linked to Proteome Discoverer v1.4 (Thermo Fisher Scientific), and the MaxQuant software v1.5.0.0. A precursor ion mass error of 5 and ,7 p.p..m respectively and a fragment ion mass error of 0.02 Da and 20 m.m.u. ,respectively were tolerated in searches of a custom made database containing the IVU proteins. GG and LRGG on lysine (K) and on serine, threonine and cysteine (S,T,C), as well as oxidation of methionine (M) were tolerated as variable modifications. Cysteine carbamidomethylation was set as a fixed modification in searches of reduced and alkylated samples. A PSM, and peptide level false discovery rate (FDR) threshold of 0.01 was applied for peptide identification employing the target-decoy database model. All ubiquitylated peptides that were also identified in IVUs lacking Ub (negative control) were discarded. Only in three cases, ubiquitylated peptides were identified in which K ubiquitylation produced the same scores as S,T,C ubiquitylation, in all other cases K ubiquitylation scored higher. Therefore in those cases when the ubiquitylation site(s) was alternatively mapped to a K or a S,T or C residue on the same peptide, S,T,C ubiquitylation was deprecated. An FDR specifically for the identification of ubiquitylated peptides was calculated. Ubiquitylated peptides in the IVUs lacking Ub (negative control) were used to model the H_0_ of random peptide spectral matching and estimate the number of false positives (FP). Ubiquitylated peptides identified in the IVUs containing Ub (supplemented with AUX/IAA or not) were used to estimate the number of true and false positives (TP+FP), because while all Ub identifications in the negative control are FP by design, only some in the IVUs containing Ub will also be FP. The number of acquired MS/MS spectra and PSMs was essentially the same for the negative control and targets (190272, 178910, 182152 MS/MS spectra and 38994, 38984, 40288 PSMs respectively) underscoring the validity of the H_0_ model. The simple FDR was calculated as FP/TP+FP. The percentage incorrect in target (FP in denominator; PIT) was estimated by determining the ratio of non-significant to total peptide identifications by the Mascot software. The simple FDR was adjusted accordingly (for further explanations see^74^). All mass spectrometry proteomics data have been deposited to the ProteomeXchange Consortium via the PRIDE^75^ partner repository (http://www.ebi.ac.uk/pride/archive/) with the data set identifier PXD004027 and 10.6019/PXD004027.

### Meta-structure analyses

Meta-structure analyses for compactness were carried out using the primary structure of IAA6 and IAA19. Plots of compactness and secondary structure are predictions based on collected pdb structures and aa contacts^76^.

### Ratiometric analysis in *Arabidopsis* protoplasts

Sensor constructs for expression in plant protoplasts were generated as described in (ref. 56). In brief, the cDNAs of IAA6, IAA19 or their dominant mutated versions iaa6/shy1-1 or iaa19/msg2-1 were amplified and Gibson cloned into the existing pMIR expression vector, where the sensor module (L2min17-Luc) was replaced. Sensors encode for renilla-2A-SM-firefly fusions under the control of a CaMV 35S promoter.

For protoplast isolation, two to three-week old plants *of A. thaliana* (*Col-0*) or *tir1-1, afb1-3, afb1-2 afb2-3, tir1-1 afb2-3, tir1-1 afb3-4* grown at a 16 h light regime at 23 °C were used. Tissue pre-plasmolysis, digestion, protoplast isolation and transformation were performed according to (ref. 77). For each ratiometric construct tested, five separate transformations with 500,000 protoplasts in a final volume of 1.6 ml were made in a six-well plate, sealed with parafilm, and incubated in the dark for 24 h. Before induction with different IAA concentrations, the replicate transformations were pooled and 1 ml of protoplast solution was transferred into a 2 ml deep-well storage plate for every auxin concentration to be tested. Serial dilutions of IAA solutions in PCA-M medium (PCA salts, 600 mOsm mannitol, pH 5.8) were prepared at 11-fold concentration, and 100 μl were added to the protoplasts to obtain the appropriate final auxin concentration. For luminescence determinations, 80 μl of protoplast suspensions of each *A. thaliana* genetic background transformed with the sensor constructs were transferred to 96-well flat-bottom white plates. After addition of 20 μl of either firefly luciferase substrate (20 mM Tricine, 2.67 mM MgSO_4_, 0.1 mM EDTA, 33.3 mM DTT, 0.52 mM ATP, 0.27 mM acetyl-CoA, 5 mM NaOH, 0.264 mM MgCO_3_, 0.47 mM luciferin), or renilla luciferase substrate (472 μM coelenterazine stock solution in methanol; diluted 1:15 in PBS directly before use). Samples were incubated in the dark for 30 min upon which firefly and renilla luminescence were monitored using either a Synergy 4 multimode microplate reader (BioTek Instruments Inc., Winooski, VT) or an Infinite M200 Pro (Tecan Group Ltd., Männedorf, Switzerland). Firefly and Renilla values for the different sensors in the different backgrounds were normalized and one- or two-way ANOVA statistical analyses were performed using RLPlot version 1.5, together with Bonferroni post-tests in GraphPad Software, Inc. Heat maps were generated in R (http://www.R-project.org/) using the gplots package with default parameters.

### Statistical analysis

Statistical analyses were performed using GraphPad Prism version 6.0, GraphPad Software, La Jolla,1 California, USA. Data were analysed by either Student's *t*-tests (two-tailed), one-way ANOVA with Tukey's honest significant difference as *post hoc* test, or two-way ANOVA with Bonferroni tests to correct for multiple testing unless otherwise stated. All experiments were repeated at least three times consisting of three-independent biological replicates. Heatmaps were generated in R (www.r-project.org) using the gplots package.

### Data availability

The authors declare that all data supporting the finding of this study are available within the article and its Supplementary Information or are available from the corresponding author upon request. Multiple sequence alignments have deposited in Figshare: https://figshare.com/s/6e202a97eb8034bbb1d9 and the mass spectrometry proteomics data have been deposited to the ProteomeXchange Consortium via the PRIDE partner repository with the data set identifier PXD004027 and Project DOI:10.6019/PXD004027.

## 

## Additional information

**How to cite this article:** Winkler, M. *et al*. Variation in auxin sensing guides AUX/IAA transcriptional repressor ubiquitylation and destruction. *Nat. Commun.* **8**, 15706 doi: 10.1038/ncomms15706 (2017).

**Publisher's note:** Springer Nature remains neutral with regard to jurisdictional claims in published maps and institutional affiliations.

## Supplementary Material

Supplementary Information Supplementary Figures, Supplementary Tables, Supplementary Note and Supplementary References

Supplementary Data 1 Sequence alignment of 29 A. thaliana AUX/IAA proteins generated for the phylogenetic tree in Supplementary Fig. 1.

Supplementary Data 2 Multiple sequence alignments of AUX/IAAs from 81 *A*. *thaliana* accessions and *A*. *halleri*, *A*. *lyrata*, and *C*. *rubella*
