# Supplementary material for: Variation in auxin sensing guides AUX/IAA transcriptional repressor ubiquitylation and destruction
Source: Nat Commun. 2017 Jun 7;8:15706. doi: 10.1038/ncomms15706 (PMC5467235; doi:10.1038/ncomms15706)
Supplement: Supplementary Information — Supplementary Figures, Supplementary Tables, Supplementary Note and Supplementary References [file ncomms15706-s1.pdf]

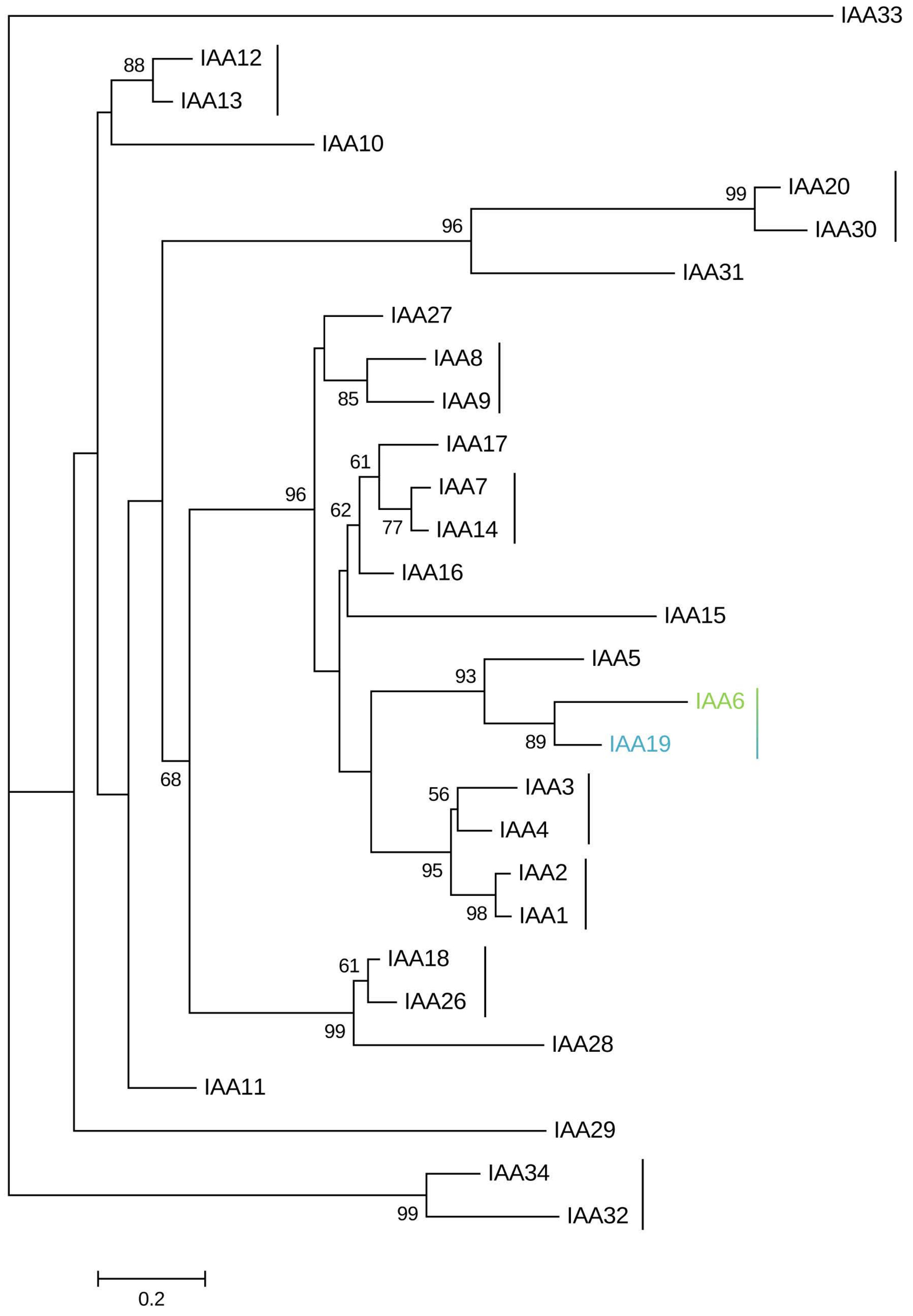


**Supplementary Figure 1.** Phylogeny of *A. thaliana* AUX/IAA proteins. The unrooted phylogenetic tree was created with the full-length protein sequences of 29 AUX/IAA proteins based on maximum likelihood. Vertical bars correspond to AUX/IAA sister pairs (ohnologs). IAA6 (green) and IAA19 (blue) ohnologs are highlighted. Bootstrap values greater than 50% are shown at the corresponding node. Scale bar 0.2 denotes 0.2 amino acid substitution per site.

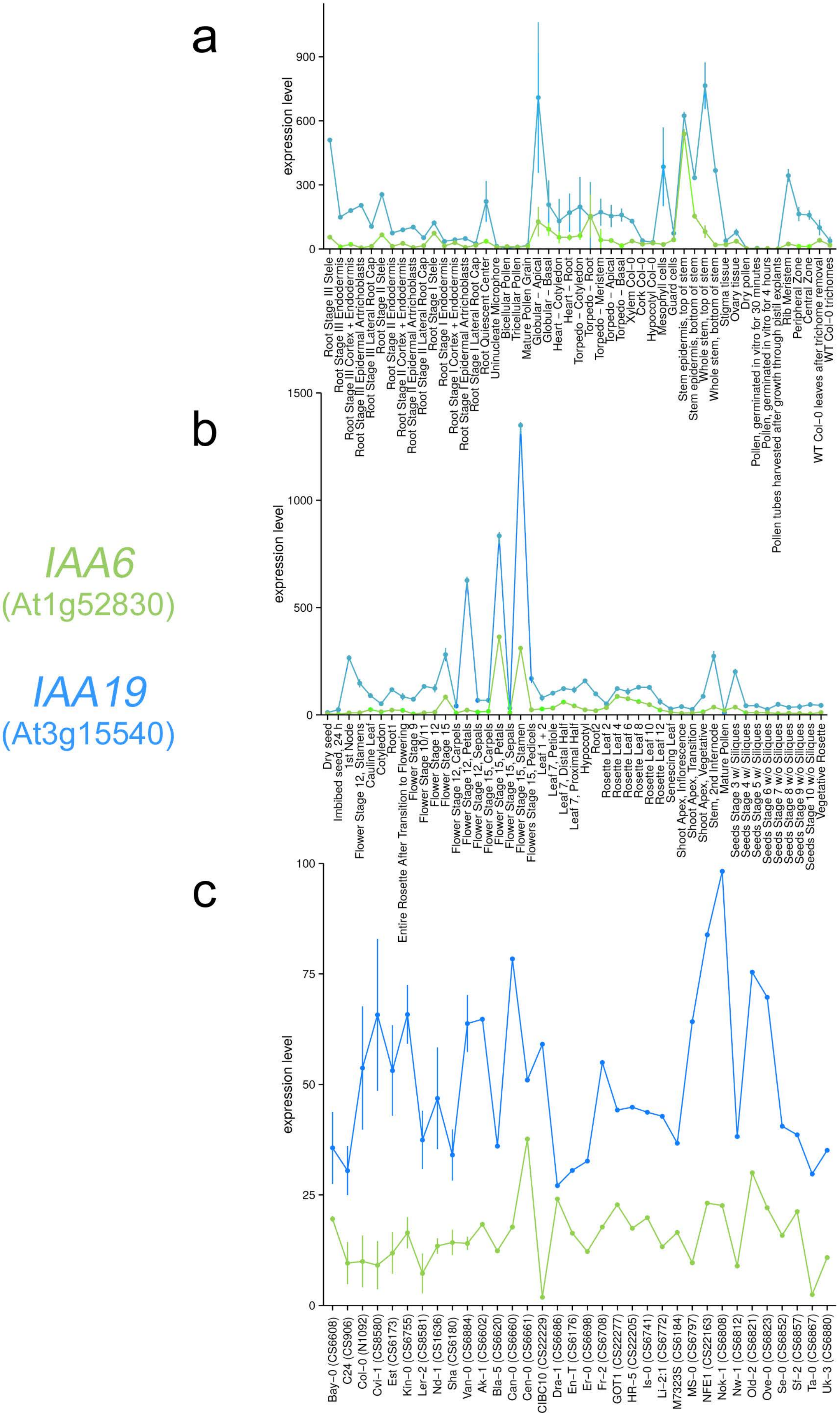


**Supplementary Figure 2.** Comparison of *IAA6* (green) and *IAA19* (blue) expression values for three different datasets: (**a**) Tissue specific/cell type, (**b**) AtGenExpress – development, and (**c**) AtGenExpress – natural variation, obtained from the Arabidopsis eFP browser (Schmidt, et al. 2005, Winter, et al. 2007). Error bars, s.d. See **Supplementary Note 1** for details and references.

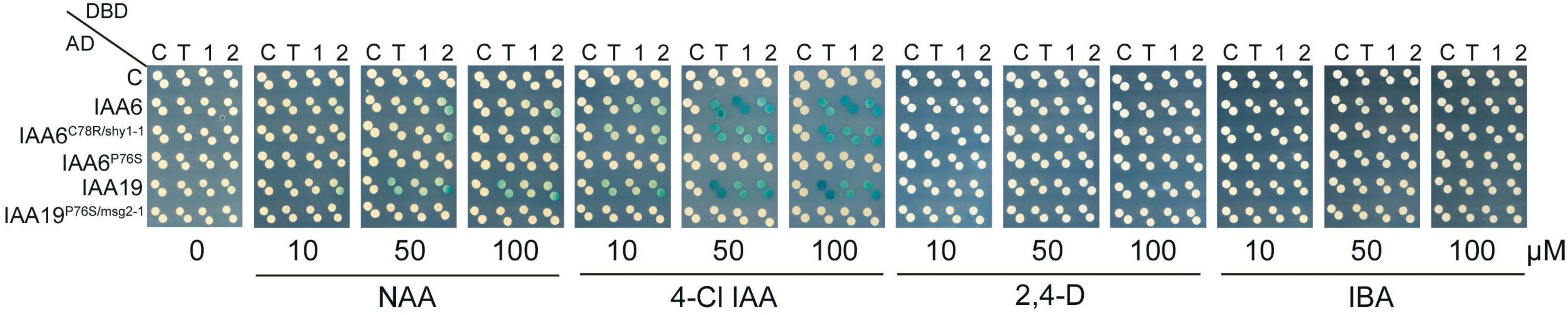


**Supplementary Figure 3**. LexA yeast two-hybrid interaction experiments of empty control (C), TIR1(T), AFB1 (1), or AFB2 (2) against IAA6, IAA6$^{C78R/shy1-1}$, IAA6$^{P76S}$, IAA19 and IAA19$^{P76S/msg2-1}$ on 10, 50 or 100 μM 1-naphthaleneacetic acid (NAA), 4-chloroindole-3-acetic acid (4-Cl IAA), 2,4-dichlorophenoxyacetic acid (2,4-D), or indole-3-butyric acid (IBA). β-galactosidase reporter expression indicates auxin-induced TIR1/AFB1/AFB2-AUX/IAA interactions.

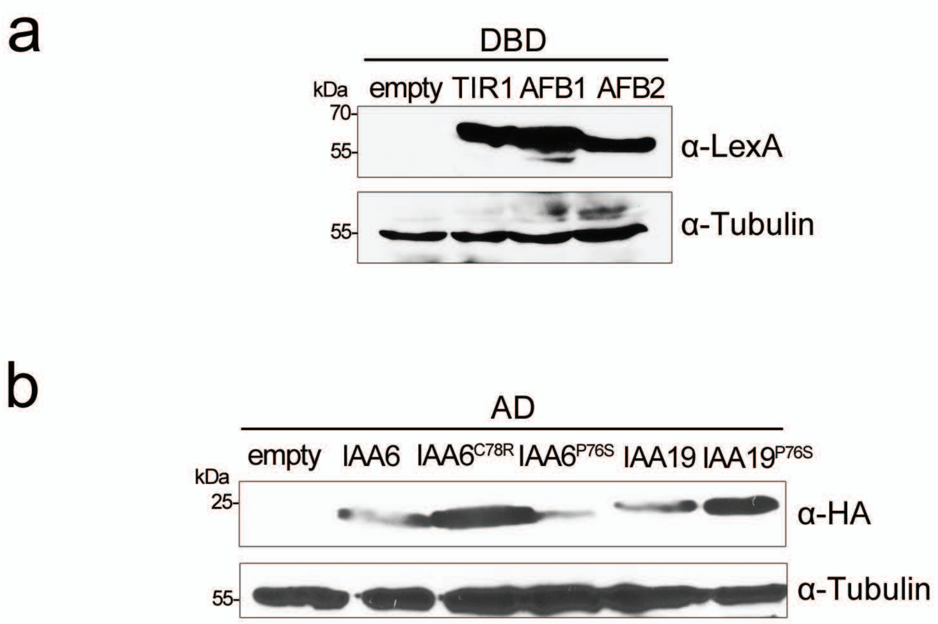


**Supplementary Figure 4**. (**a**) Immunoblots of LexA tagged -TIR1, -AFB1, -AFB2; and (**b**) HA tagged -IAA6, -IAA6$^{C78R/shy1-1}$, -IAA6$^{P76S}$, -IAA19, and -IAA19$^{P76S/msg2-1}$ proteins. TIR1, AFB1, AFB2, as well as AUX/IAA fusion proteins screened for auxin dependent interactions in yeast diploids are well expressed, and repetitive experiments (n>4) consistently showed that interactions (see **Fig. 1c and Supplementary Fig. 3**) are unlikely to be dependent upon fusion protein expression levels in yeast. Total proteins were extracted from yeast diploids grown on Gal/Raff -Ura-His-Trp and detected using either anti-HA (F-7) (Santa Cruz, SC7392) or anti-LexA (Abcam, AB14553) antibodies. Yeast α-Tubulin was detected with anti-Tubulin antibody (Abcam, AB6160) and used as loading control.

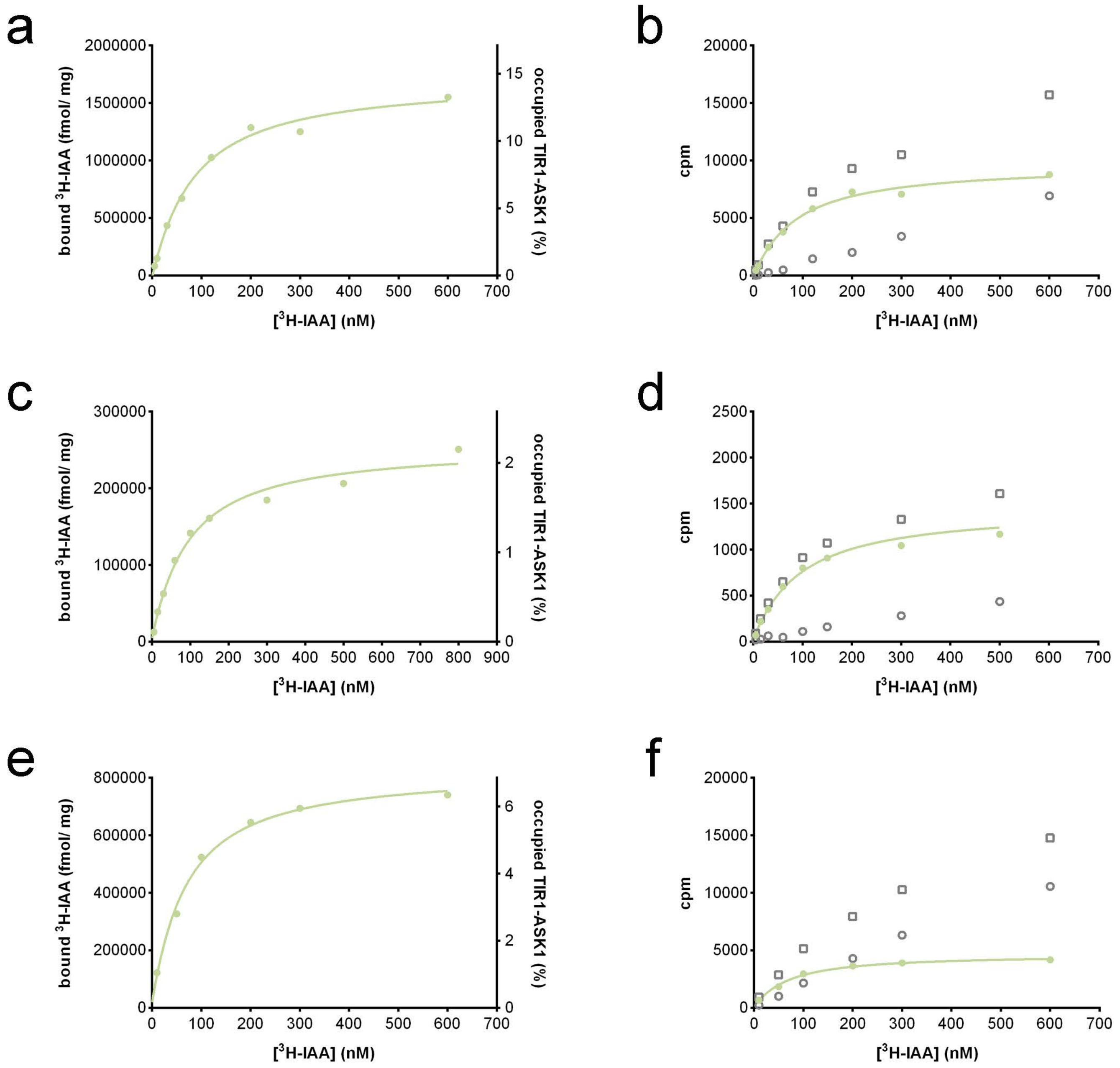


**Supplementary Figure 5**. Individual saturation binding experiments for the TIR1-IAA6 auxin co-receptor system. (**a, c, e**) Specific binding of IAA to TIR1-ASK1, expressed as either femtomol IAA bound per one milligram TIR1-ASK1 (left Y-axis) or fraction of TIR1 stably binding IAA (right Y-axis), was calculated assuming a 60% efficiency of the liquid scintillation counter and a molecular weight of 85.0 kDa for TIR1-ASK1. (**b, d, f**) Total, non-specific and specific binding (empty squares, empty circles and dots) shown as mean raw cpm data from two technical replicates. Specific binding was fitted to the Morrison model (**a-f**). Obtained $K_d$ values are (74.0 ± 19.2) nM (**a, b**), (83.7 ± 18.3) nM (**c, d**) and (58.2 ± 13.6) nM (**e, f**).

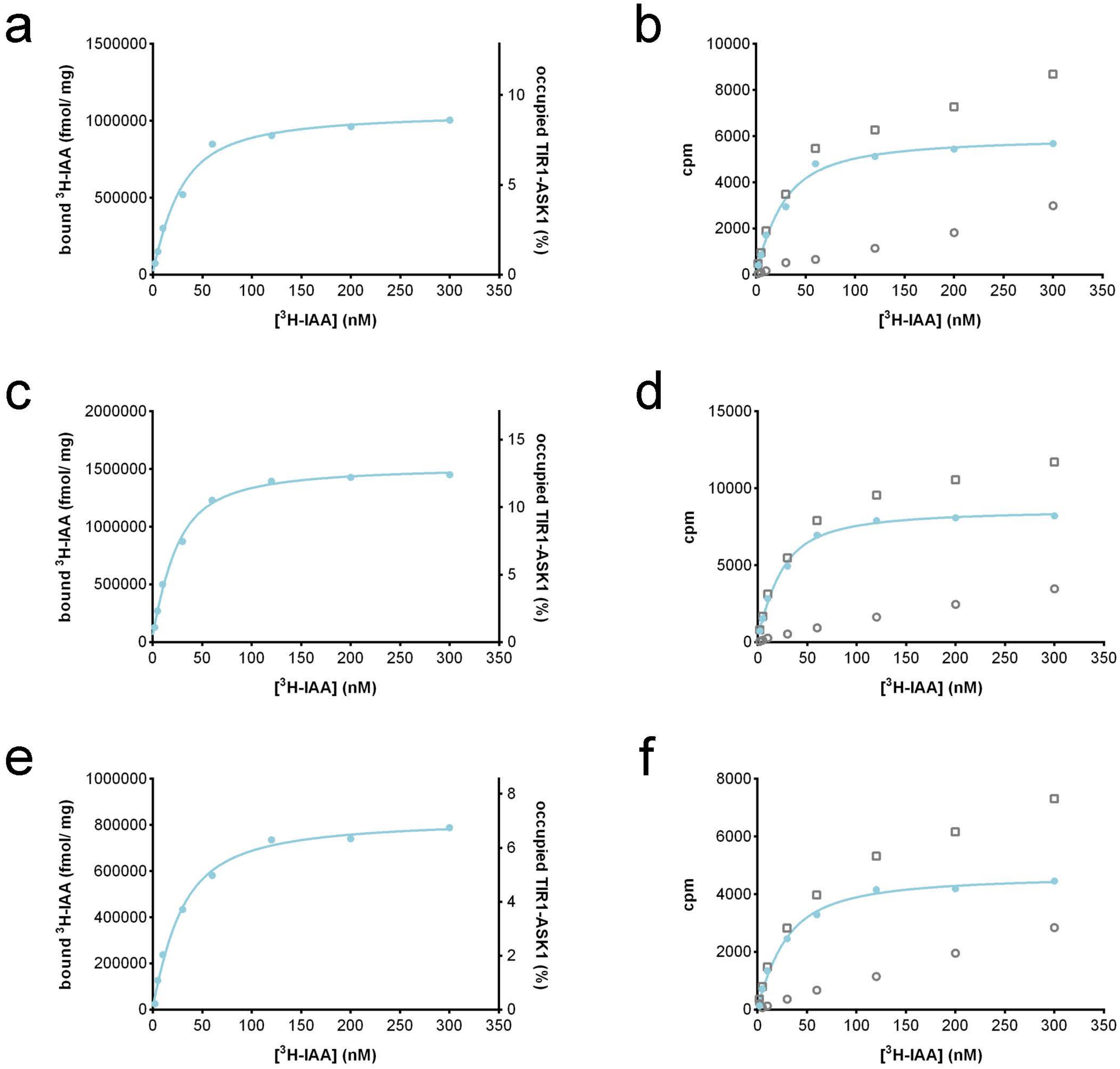


**Supplementary Figure 6.** Individual saturation binding experiments for the TIR1-IAA19 auxin co-receptor system. (**a, c, e**) Specific binding of IAA to TIR1-ASK1, expressed as either femtomol IAA bound per one milligram TIR1-ASK1 (left Y-axis), or fraction of TIR1 stably binding IAA (right Y-axis), was calculated assuming a 60% efficiency of the liquid scintillation counter and a molecular weight of 85.0 kDa for TIR1-ASK1. (**b, d, f**) Total, non-specific and specific binding (empty squares, empty circles and dots, respectively) shown as mean raw cpm data from two technical replicates. Specific binding was fitted to the Morrison model (**a-f**). Obtained $K_d$ values are (16.4 ± 4.4) nM (**a, b**), (12.8 ± 3.0) nM (**c, d**) and (17.6 ± 2.9) nM (**e, f**).

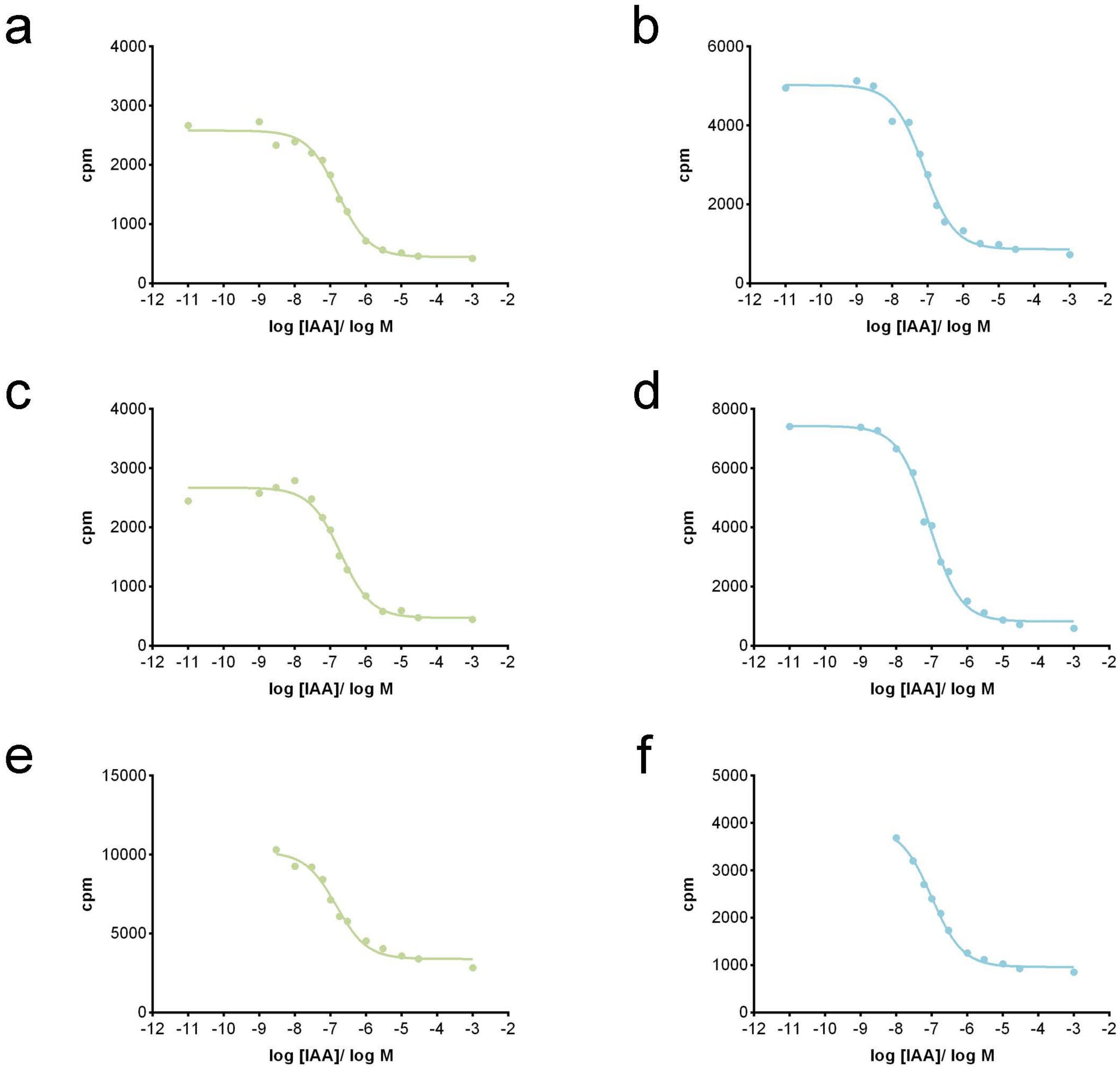


**Supplementary Figure 7**. Individual homologous competition binding experiments for TIR1-IAA6 and TIR1-IAA19 auxin co-receptors. Non-radioactive IAA was titrated to pre-formed TIR1-IAA6 (**a, c, e**) or TIR1-IAA19 (**b, d, f**) co-receptors bound to radioactive IAA. Resulting data are depicted as mean raw cpm data from two technical replicates were fitted to the built-in one site - log$IC_{50}$ equation (GraphPad, Prism). $K_i$ values were calculated according to the Cheng-Prusoff equation. $K_i$ values for TIR1-IAA6 (**a,c,e**), and TIR1-IAA19 (**b,d,f**) are: 98.0 nM (**a**), 114.5 nM (**c**), 85.4 nM (**e**); and 29.7 nM (**b**), 32.3 nM (**d**), 38.5 nM (**f**).

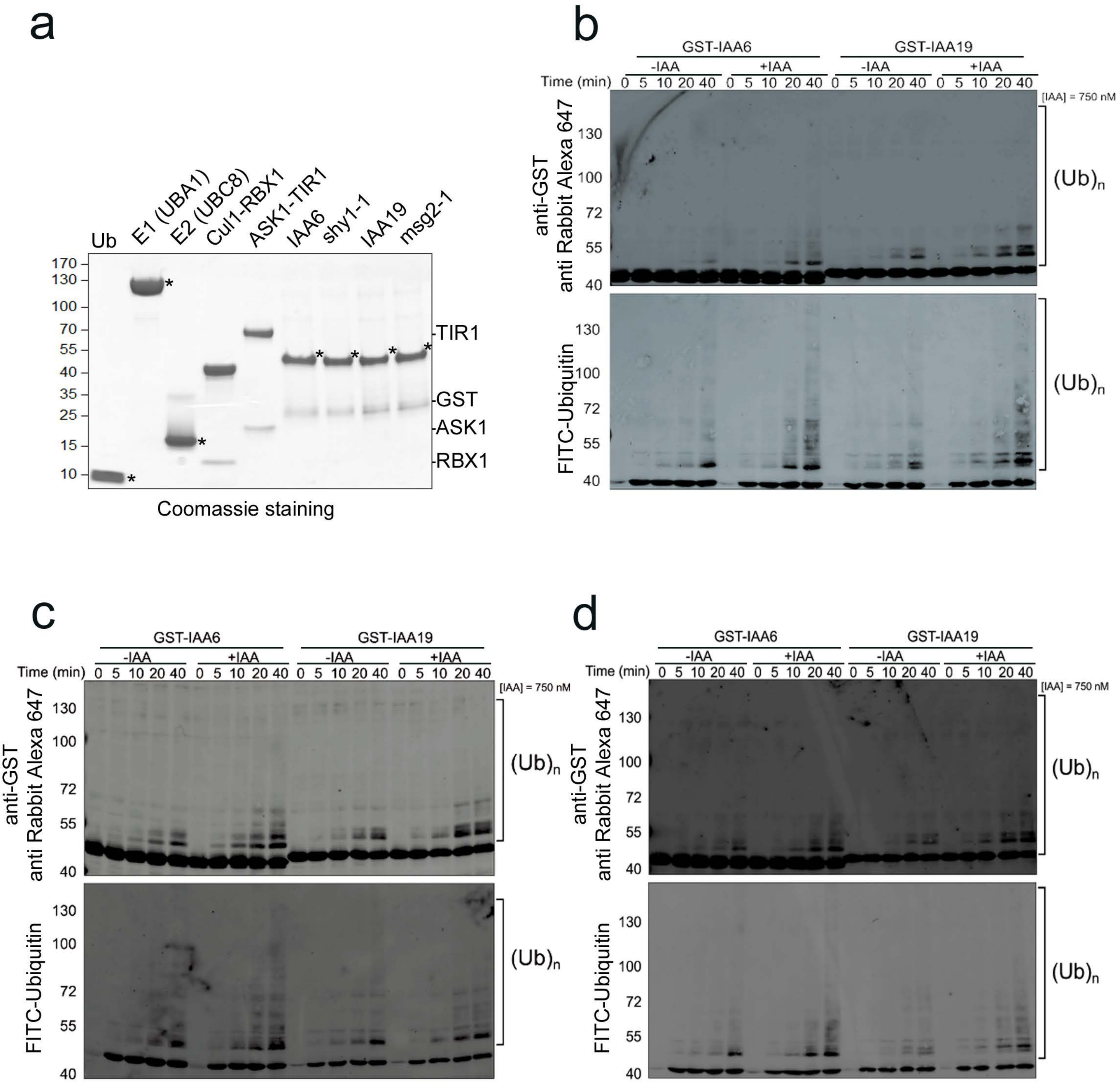


**Supplementary Figure 8.** (**a**) Coomassie staining of an SDS-gradient 4-12% gel depicting quality of proteins (~5 µg) incorporated in IVU reactions. (**b-d**) Replicate blots from 3 independent IVUs used for quantification in **Figure 2c**. Fluorescein-labeled ubiquitin signals of individual lanes were quantified using ImageQuant TL software. Shown are immunoblots for either direct ubiquitin detection (bottom) or AUX/IAA detection using α-GST and α-rabbit Alexa Fluor Plus 647 antibodies (see **Methods** for additional information). (**d**) Immunoblots correspond to **Figure 2c** in gray scale. (**e and f**) continued in next page.

e

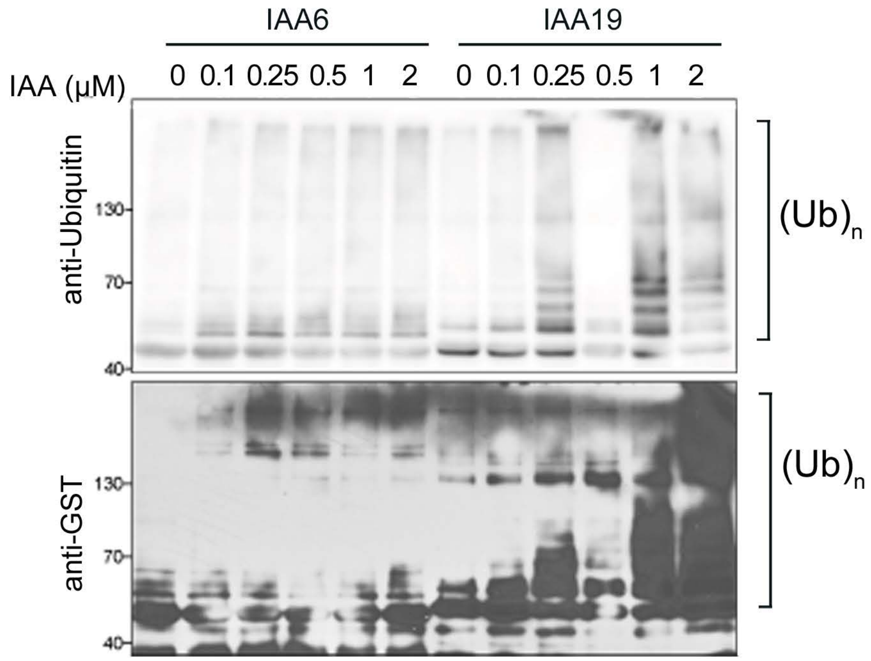


f

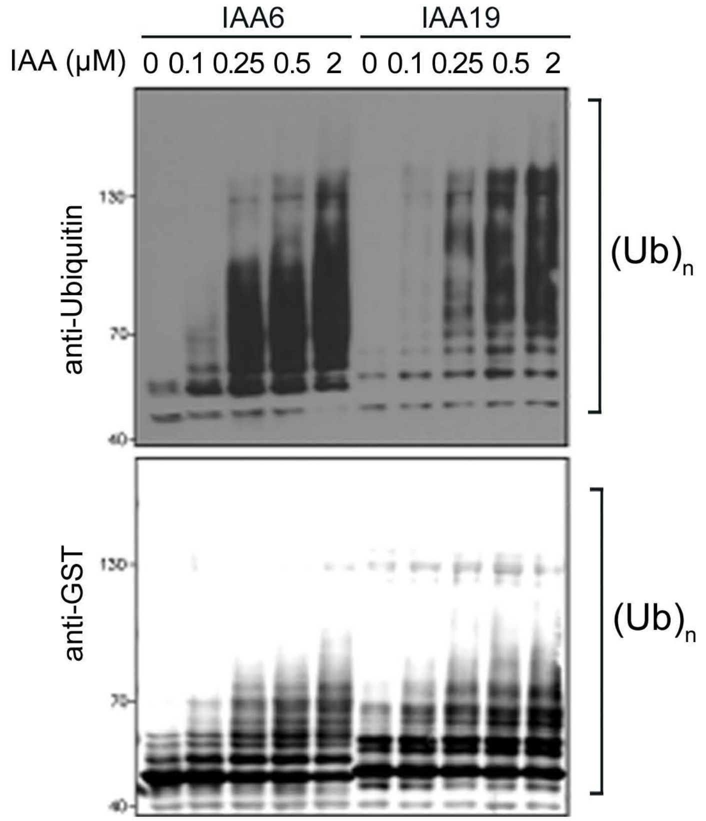


**Supplementary Figure 8** continued. (**e and f**) Replicates showing auxin induced ubiquitylation of GST-IAA6 and GST-IAA19 (**see Figure 2d**). AUX/IAA ubiquitylated species were detected using α-Ub (P4D1) antibody and α-GST antibody.

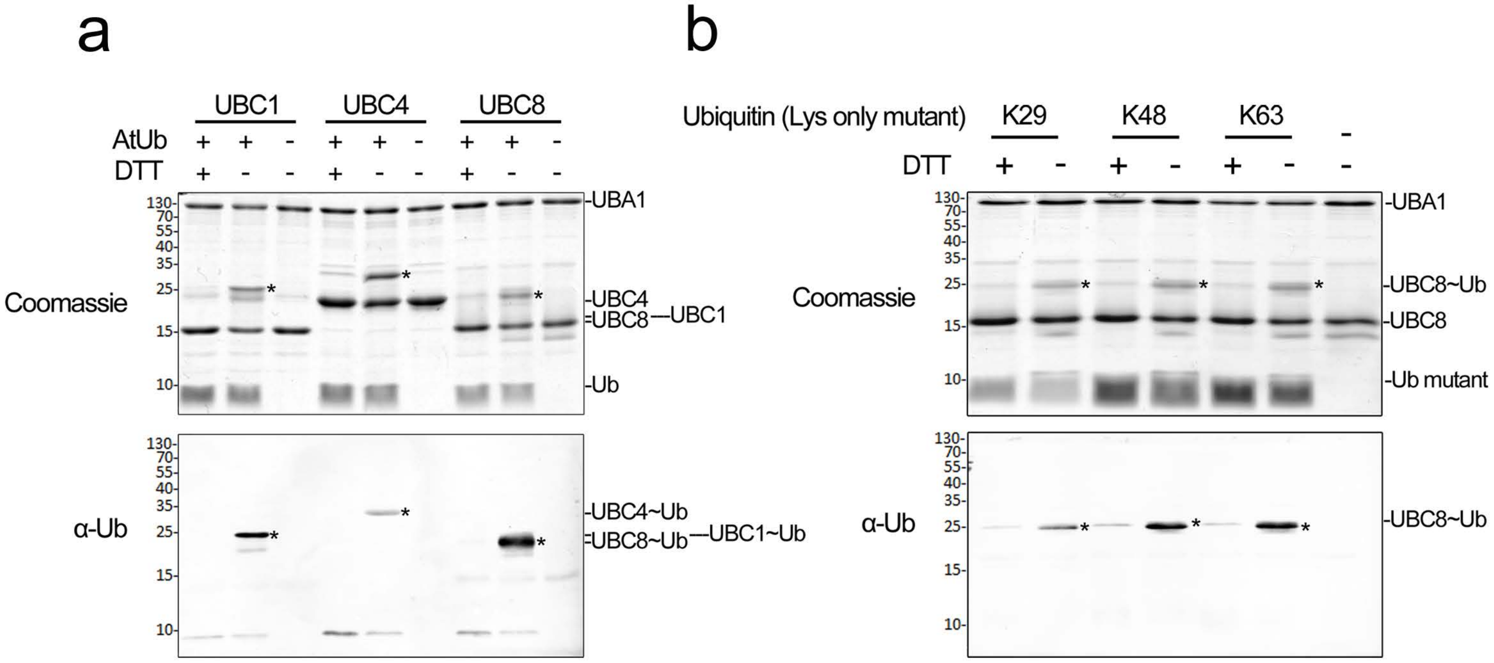


**Supplementary Figure 9**. ATP-dependent ubiquitin charging assays of Arabidopsis E2-ubiquitin conjugating enzymes used in this study. (**a**) UBC1, UBC4, and UBC8 charging assays show recombinantly expressed AtE2s are functional and can be Ub-charged *in vitro*. Asterisks indicate Ub-charged E2s (UBCx~Ub). (**b**) UBC8 can form a thioester bond with *Hs*Ubiquitin mutants containing only one lysine (Lys) residue available, namely K29, K48 or K63, while other Lys have been mutated to Arg. E2 (UBC8)-charging reactions were performed as described in the **Methods** section.

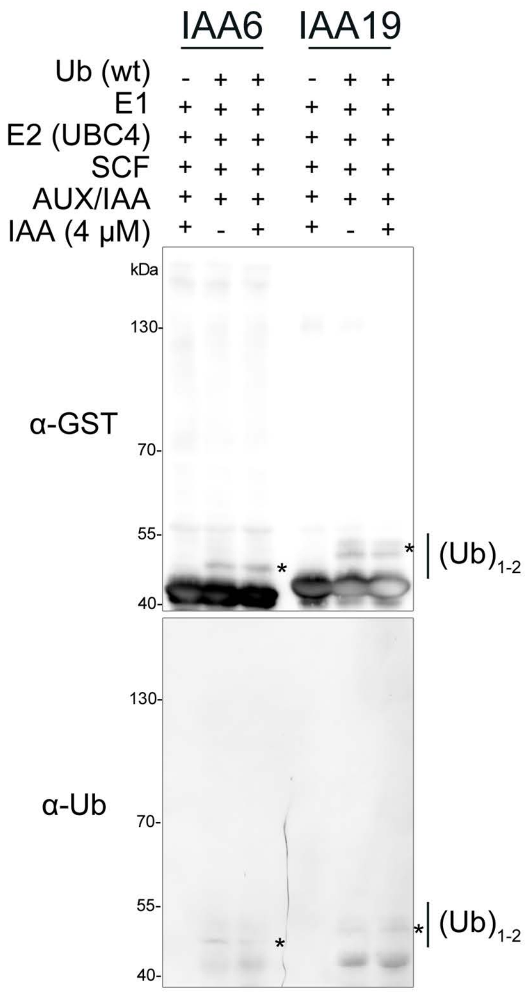


**Supplementary Figure 10**. UBC4 Ub-conjugating enzyme (E2) does not mediate poly-Ub-conjugation of IAA6 or IAA19 *in vitro*. As a result of 30 min IVU assays for IAA6 and IAA19, only low molecular ubiquitin conjugates, possibly monoubiquitin or diubiquitin ($Ub_{1-2}$), have been consistently detected with anti-GST and anti-Ub antibodies (*).

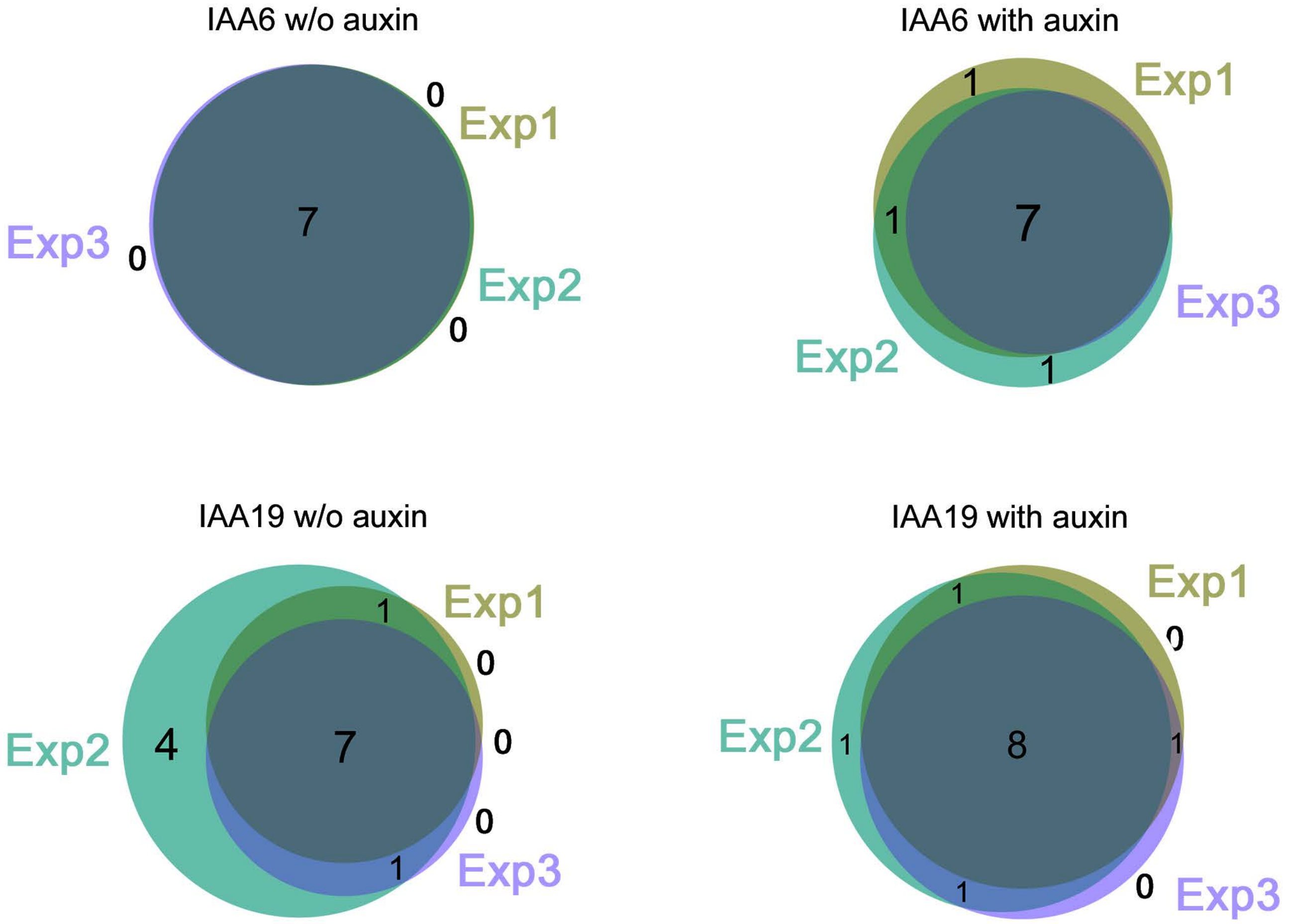


**Supplementary Figure 11**. Venn diagrams indicating the reproducibility in the identification of IAA6 and IAA19 ubiquitylated peptides upon three independent biological IVU reactions (Exp 1-3) with or without auxin followed by LC-MS analyses.

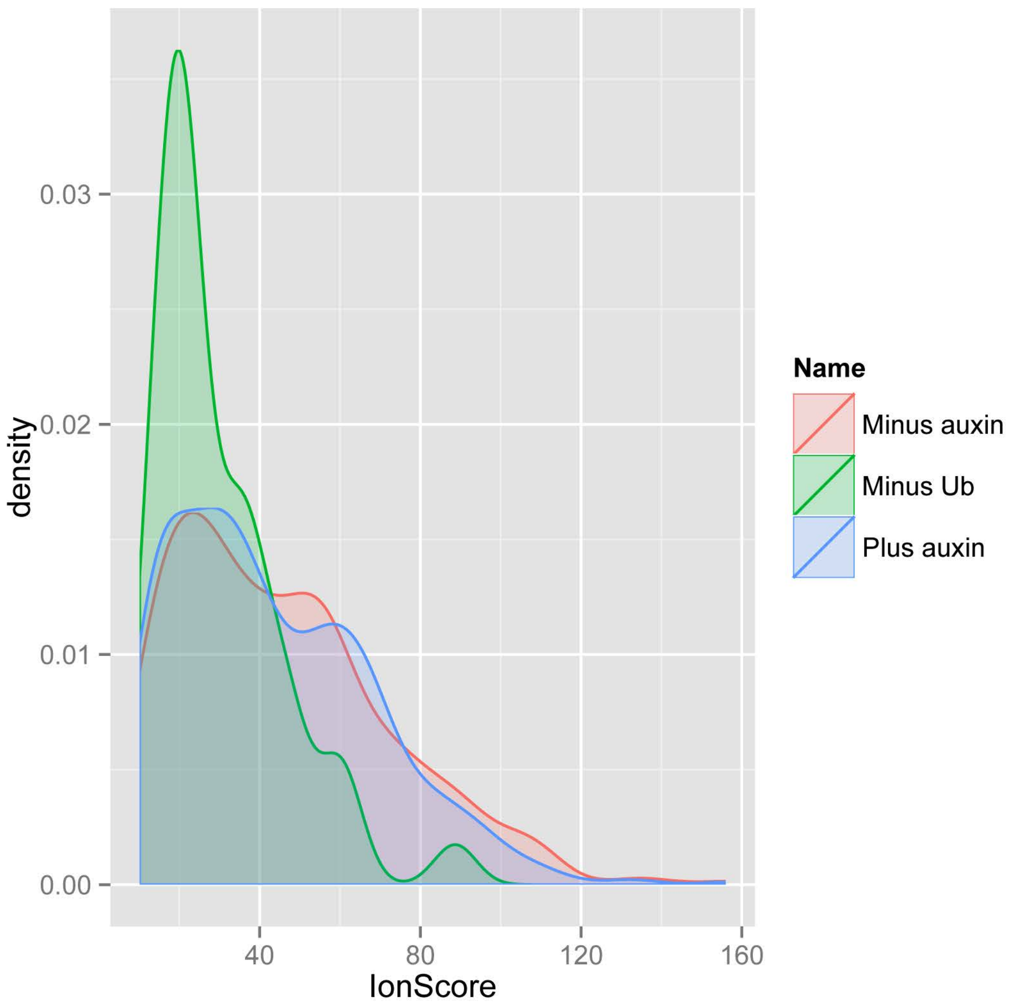


**Supplementary Figure 12.** Calculated false discovery rate (FDR) for IAA6 or IAA19 ubiquitylated peptides identified via LC-MS. Kernel density estimation of the PSMs (see **Supplementary Tables 1-2**) with a GG- modification identified in IVUs lacking Ub (Minus Ub), or containing Ub (supplemented with AUX/IAA or not, Plus Auxin, Minus Auxin respectively). FDR was calculated as described in the Methods section. An ion score of below 20 corresponded to an FDR threshold of 0.05 ($q<0.05$) for IVUs containing Ub and supplemented with auxin. The same ion score corresponded to an FDR threshold of 0.07 for IVUs containing Ub and not supplemented with auxin.

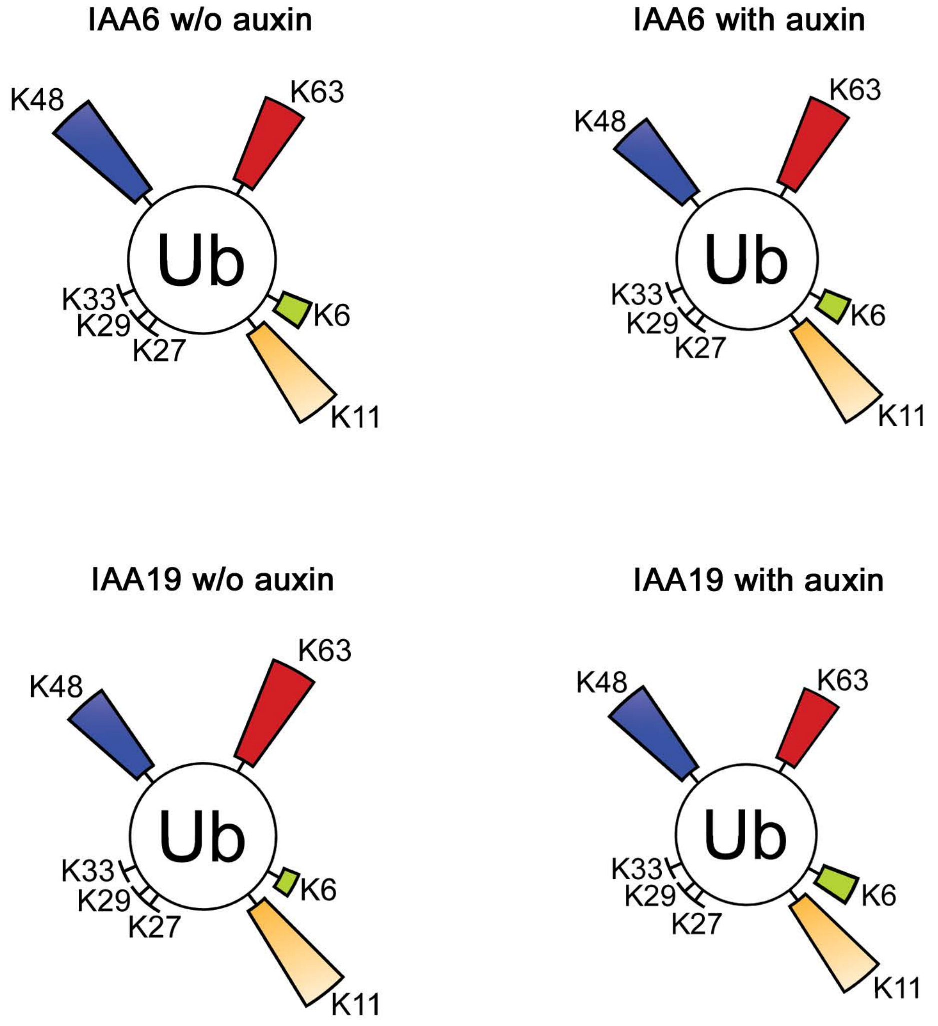


**Supplementary Figure 13.** Distribution of identified ubiquitin linkage types. *In vitro* ubiquitylation reactions for IAA6 (top) and IAA19 (bottom) were analyzed via LC-MS, and ubiquitin peptides corresponding to different ubiquitin linkage types were identified. A comparison of the number of peptide spectrum matches (PSMs) (see **Supplementary Tables 1-2** and PRIDE repository via ProteomeXchange with identifier PXD004027) for each of the ubiquitylated lysine residues in ubiquitin was used as a semi-quantitative index for determining the abundance of each linkage type in the reactions without or with auxin (4 μM IAA). Depicted values for a specific linkage type were calculated as the percentage of the total number of PSMs corresponding to diGly-/LRGG-modified ubiquitin peptides. All peptides assigned to contain the Ub remnant (diGly or LRGG) on non-canonical residues were excluded. No K27, K29 nor K33 linkages were detected in these reactions using UBC8 as E2-ubiquitin conjugating enzyme.

a

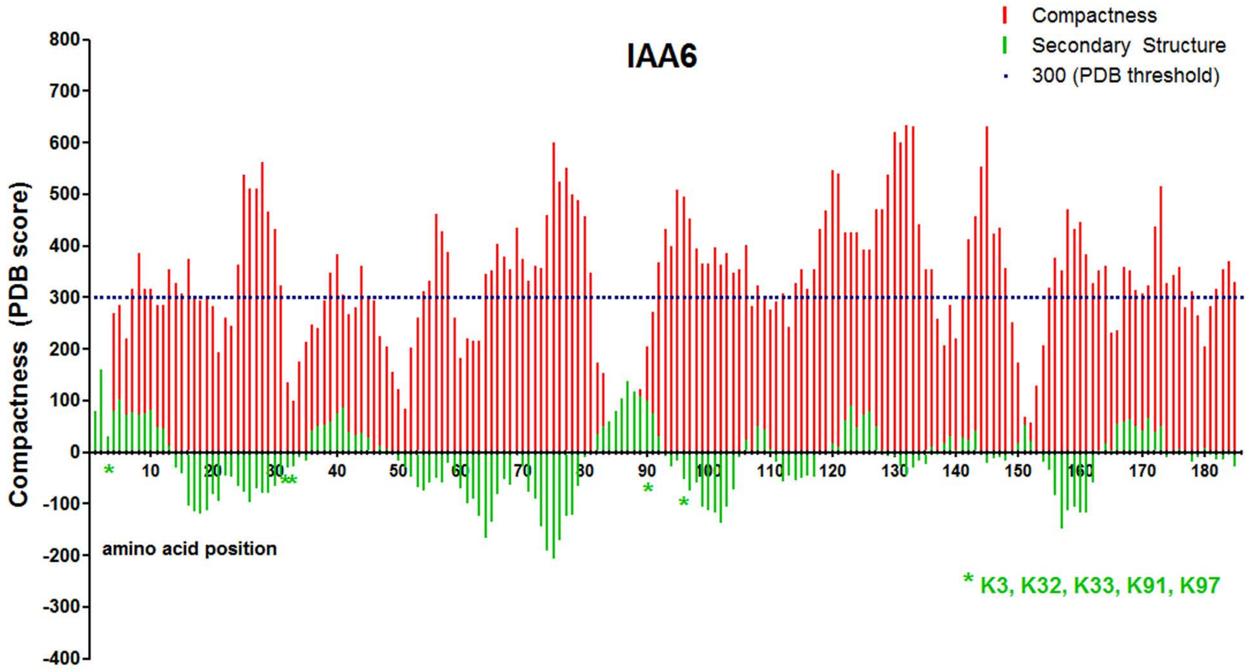


b

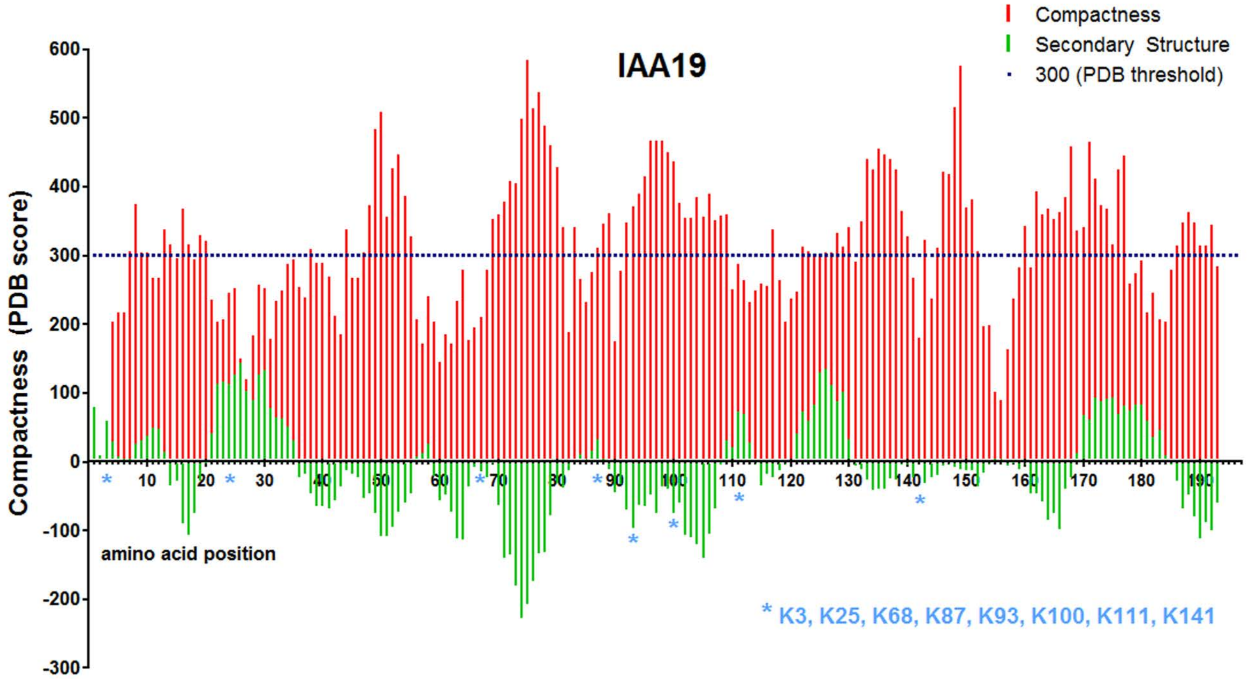


**Supplementary Figure 14. (a-b)** Meta structure analyses for Arabidopsis IAA6 **(a)** and IAA19 **(b)**, exhibiting features of intrinsically disordered proteins (IDPs) with regions of lower compactness. The threshold level in compactness is 300 (DisProt data base), while sequence regions that are significantly lower are flexible and open, amino acid segments above threshold can be considered classically folded. In secondary structure positive values denote alpha-helical regions, negative values beta sheets. Asterisks depict ubiquitylated sites identified via MS upon IVU reactions (for reference see **Supplementary Tables 1-2**).

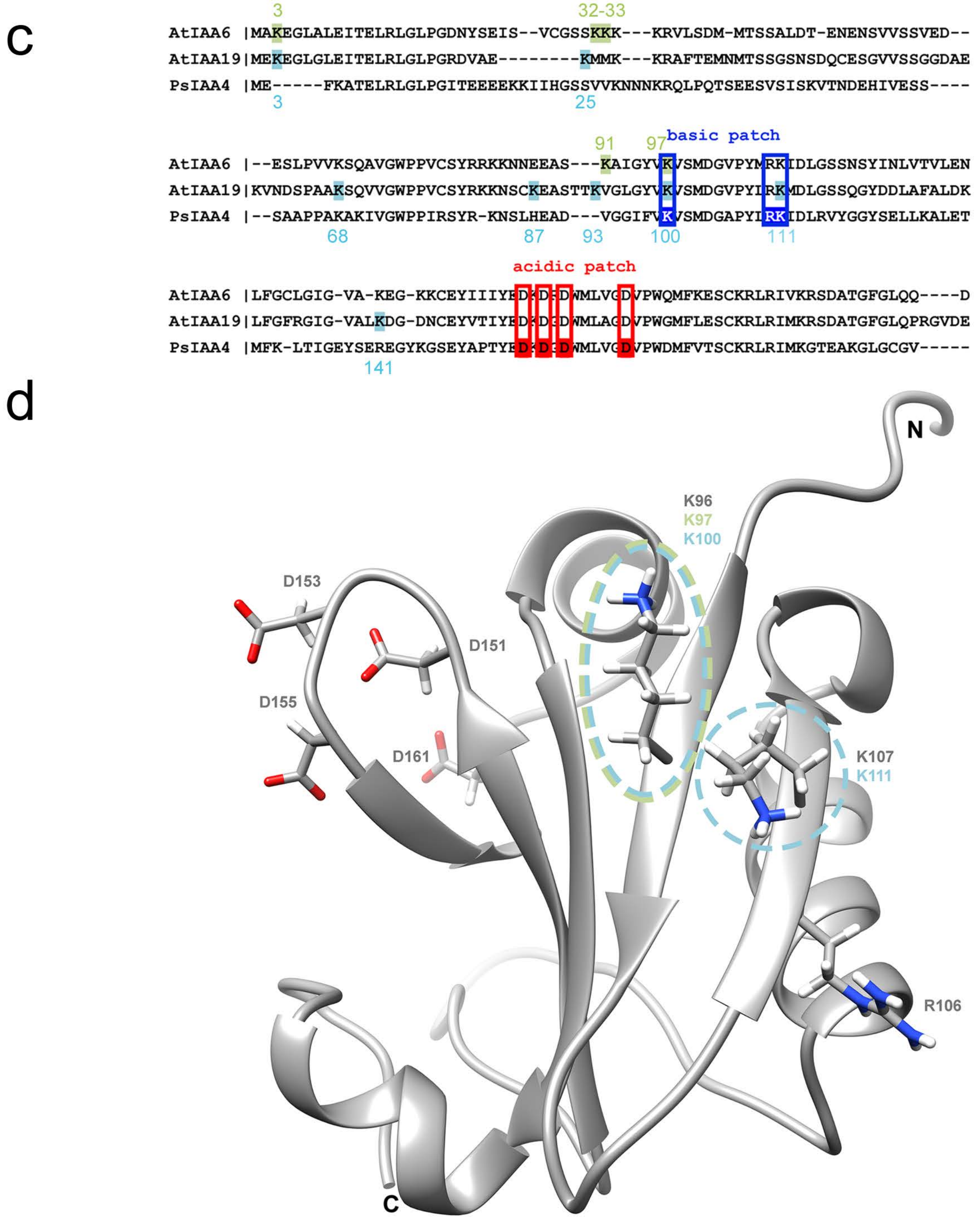


**Supplementary Figure 14** continued. **(c-d)** Interface residues crucial for AUX/IAA and ARF homo- and hetero-dimerization are targeted for ubiquitylation *in vitro*. (**c**) MAFFT alignment of *A. thaliana At*IAA6, *At*IAA19 and *Pisum sativum Ps*IAA4. IAA6 and IAA19 residues found to be ubiquitylated by UBC8 in vitro are highlighted in green and blue, respectively. K97 of IAA6, and K100 as well as K111 of IAA19 are part of the basic patch in the AUX/IAA PB1 domain (Dinesh et al. PNAS 2015). (**d**) Structural representation of the acidic (D151, D153, D155, D161) and basic (K96, R106, K107) patches in the PB1 domain of PsIAA4, PDB: 2M1M). Ubiquitylation of K97 of IAA6 (green ellipse) and K100 and K111 of IAA19 (blue ellipses) could interfere with proper dimerization of IAA6 and IAA19 with other AUX/IAAs and/or ARFs.

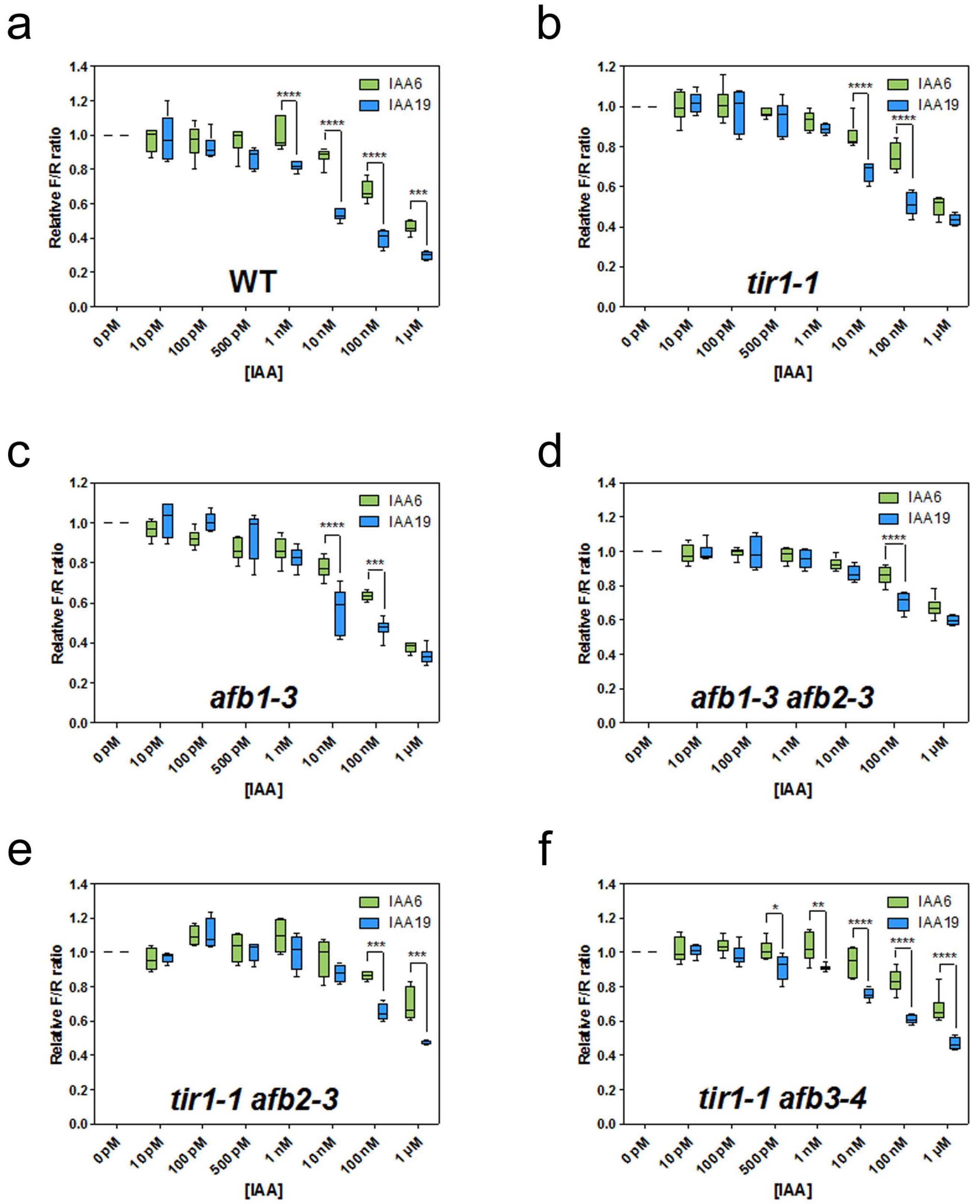


**Supplementary Figure 15**. Quantitative characterization of IAA6 and IAA19 sensor variants in different genetic backgrounds of *Arabidopsis* leaf protoplasts, namely from *Col-0 (WT)* (**a**), as well as *tir1-1* (**b**), *afb1-3* (**c**), *afb1-3 afb2-3* (**d**), *tir1-1 afb2-3* (**e**), and *tir1-1 afb3-4* (**f**) mutant plants. Decrease in F/R ratios is a measure of sensor degradation after 30 min incubation with various IAA concentrations. Results are means +/- s.e.m. of biological replicates (n=6). Statistical significances were calculated via two-way ANOVA with P<0.05 (*), P<0.01(**), P<0.001 (***), and P<0.0001 (****).

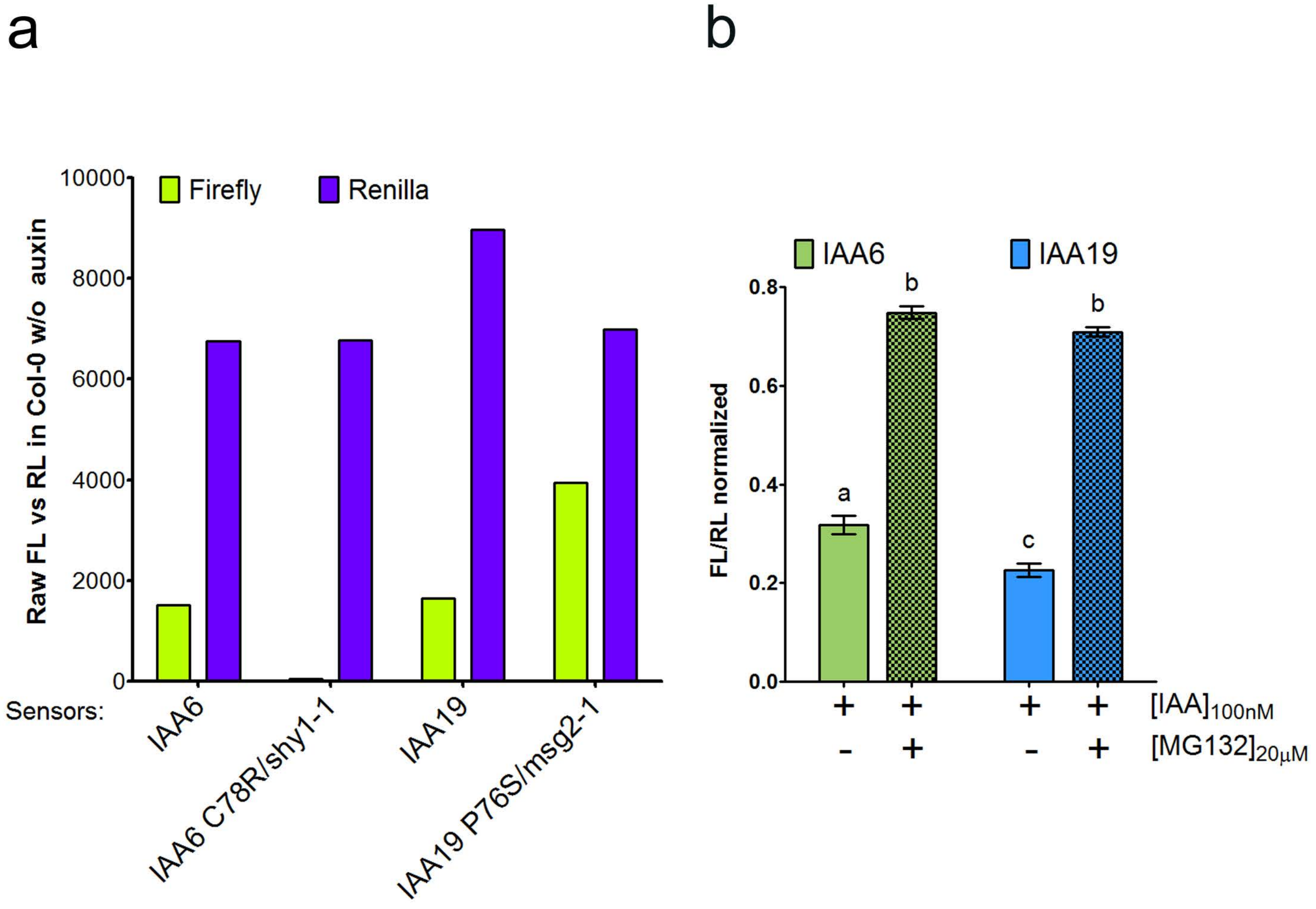


**Supplementary Figure 16.** (**a**) Raw values of Firefly luciferase (FL) -fused IAA6, IAA6$^{C78R/shy1-1}$, IAA19, IAA19$^{P76S/msg2-1}$ sensors and Renilla luciferase (RL) fluorescence of the normalization element in *Col-0* protoplasts without exogenous IAA treatment. While IAA6, IAA19, and IAA19$^{P76S/msg2-1}$ sensors are similarly expressed, IAA6$^{C78R/shy1-1}$ sensor is highly unstable and not suitable for ratiometric analyses. (**b**) Ratiometric analysis of IAA6 and IAA19 sensors in protoplasts of *Col-0 (WT)* plants are degraded in response to 100 nM IAA and stabilized upon combined treatment of IAA and 20 μM proteasome inhibitor MG132. Error bars, s.e.m. Different letters denote statistically significant differences as assessed by two-way ANOVA with P<0.001, P>0.05 (n.s.).

## Supplementary Table 1. IAA6 ubiquitylated peptides identified in LC-MS analyses

### IAA6 w/o Auxin

| | | Mascot | | | MaxQuant | | | | | | | |
|---|---|---|---|---|---|---|---|---|---|---|---|---|
| Sequence | # PSMs | Modifications | IonScore | Exp Value | Modifications | Score | PEP* | MH+ [Da] | Charge | m/z [Da] | RT | Exp |
| AGFMAKEGLALEITELR | 6 | K6(di-Gl) | 78 | 3.14019E-07 | K6(di-Gl) | 101 | 1.53E-16 | 1963.0252 | 3 | 655.0132 | 67.64 | 1 |
| AIGYVKVSMDGVPYMR | 1 | K6(di-Gl) | 58 | 3.29577E-05 | K6(di-Gl) | 69 | 3.50E-07 | 1899.9508 | 3 | 633.9885 | 57.37 | 1 |
| KAGFMAKEGLALEITELR | 2 | K7(di-Gl) | 26 | 0.044766853 | K7(di-Gl) | 43 | 7.78E-05 | 2091.1251 | 4 | 523.5367 | 57.96 | 1 |
| LGLPGDNYSEISVCGSSKK | 4 | C14(Carbamidomethyl); K19(di-Gl) | 77 | 3.82786E-07 | C14(Carbamidomethyl); K18(di-Gl) | 71 | 2.88E-24 | 2125.0210 | 2 | 1063.0142 | 47.49 | 1 |
| LGLPGDNYSEISVCGSSKKK | 6 | C14(Carbamidomethyl); K20(di-Gl) | 37 | 0.003597134 | C14(Carbamidomethyl); K18(di-Gl) | 83 | 5.54E-27 | 2253.1139 | 3 | 751.7130 | 41.33 | 1 |
| NNEEASKAIGYVK | 3 | K7(di-Gl) | 92 | 1.31207E-08 | K7(di-Gl) | 102 | 4.29E-14 | 1536.7666 | 2 | 768.8856 | 30.79 | 1 |
| AGFMAKEGLALEITELR | 4 | K6(di-Gl) | 78 | 2.99886E-07 | K6(di-Gl) | 108 | 2.79E-18 | 1963.0272 | 3 | 655.0139 | 67.44 | 2 |
| AIGYVKVSMDGVPYMR | 1 | K6(di-Gl) | 46 | 0.00055457 | K6(di-Gl) | 50 | 0.00012455 | 1899.9364 | 3 | 633.9836 | 57.71 | 2 |
| KAGFMAKEGLALEITELR | 1 | K7(di-Gl) | 14 | 0.856952141 | n.d. | n.d. | n.d. | 2091.1228 | 4 | 523.5362 | 58.53 | 2 |
| LGLPGDNYSEISVCGSSKK | 4 | C14(Carbamidomethyl); K19(di-Gl) | 105 | 6.20807E-10 | C14(Carbamidomethyl); K18(di-Gl) | 104 | 5.47E-37 | 2125.0191 | 2 | 1063.0132 | 48.02 | 2 |
| LGLPGDNYSEISVCGSSKK | 2 | C14(Carbamidomethyl); K18(LRGG) | 19 | 0.279226459 | n.d. | n.d. | n.d. | 2394.2069 | 4 | 599.3072 | 43.57 | 2 |
| LGLPGDNYSEISVCGSSKKK | 3 | C14(Carbamidomethyl); K18(di-Gl); K19(di-Gl) | 21 | 0.155223177 | C14(Carbamidomethyl); K18(di-Gl); K19(di-Gl) | 43 | 7.86E-17 | 2367.1559 | 4 | 592.5444 | 41.21 | 2 |
| LGLPGDNYSEISVCGSSKKK | 6 | C14(Carbamidomethyl); K18(di-Gl) | 48 | 0.000305462 | C14(Carbamidomethyl); K18(di-Gl) | 83 | 7.86E-17 | 2253.1149 | 3 | 751.7099 | 41.27 | 2 |
| NNEEASKAIGYVK | 3 | K7(di-Gl) | 92 | 1.30004E-08 | K7(di-Gl) | 101 | 2.05E-13 | 1536.7624 | 2 | 768.8840 | 31.13 | 2 |
| AGFMAKEGLALEITELR | 4 | K6(di-Gl) | 72 | 1.34573E-06 | K6(di-Gl) | 63 | 5.08E-07 | 1963.0292 | 3 | 655.0146 | 68.24 | 3 |
| AIGYVKVSMDGVPYMR | 1 | K6(di-Gl) | 29 | 0.02582002 | K6(di-Gl) | 69 | 3.92E-07 | 1899.9406 | 3 | 633.9850 | 58.05 | 3 |
| KAGFMAKEGLALEITELR | 2 | K7(di-Gl) | 25 | 0.069655685 | K7(di-Gl) | n.d. | n.d. | 2091.1278 | 4 | 523.5374 | 58.55 | 3 |
| LGLPGDNYSEISVCGSSKK | 4 | C14(Carbamidomethyl); K19(di-Gl) | 83 | 9.54897E-08 | C14(Carbamidomethyl); K18(di-Gl) | 92 | 1.94E-16 | 2125.0210 | 3 | 709.0118 | 48.10 | 3 |
| LGLPGDNYSEISVCGSSKK | 2 | C14(Carbamidomethyl); K18(LRGG) | 38 | 0.003012705 | C14(Carbamidomethyl); K18(LRGG) | 62 | 1.94E-16 | 2394.2085 | 3 | 798.7410 | 43.84 | 3 |
| LGLPGDNYSEISVCGSSKKK | 6 | C14(Carbamidomethyl); K18(di-Gl) | 45 | 0.000642623 | C14(Carbamidomethyl); K18(di-Gl) | 65 | 5.26E-20 | 2253.1203 | 3 | 751.7128 | 41.66 | 3 |
| LGLPGDNYSEISVCGSSKKK | 2 | C14(Carbamidomethyl); K18(di-Gl); K19(di-Gl) | 16 | 0.519943997 | C14(Carbamidomethyl); K18(di-Gl); K19(di-Gl) | 42 | 5.26E-20 | 2367.1513 | 4 | 592.5433 | 41.57 | 3 |
| NNEEASKAIGYVK | 2 | K7(di-Gl) | 92 | 1.23867E-08 | K7(di-Gl) | 122 | 9.80E-13 | 1536.7674 | 2 | 768.8873 | 31.29 | 3 |

### IAA6 with 4 μM IAA

| | | Mascot | | | MaxQuant | | | | | | | |
|---|---|---|---|---|---|---|---|---|---|---|---|---|
| Sequence | # PSMs | Modifications | IonScore | Exp Value | Modifications | Score | PEP* | MH+ [Da] | Charge | m/z [Da] | RT | Exp |
| AGFMAKEGLALEITELR | 7 | K6(di-Gl) | 83 | 9.95306E-08 | K6(di-Gl) | 106 | 2.77E-16 | 1963.0272 | 3 | 655.0139 | 71.26 | 1 |
| AIGYVKVSMDGVPYMR | 1 | K6(di-Gl) | 62 | 1.2559E-05 | K6(di-Gl) | 77 | 5.87E-07 | 1899.9461 | 3 | 633.9869 | 57.51 | 1 |
| KAGFMAKEGLALEITELR | 2 | K7(di-Gl) | 30 | 0.019676895 | n.d. | n.d. | n.d. | 2091.1239 | 4 | 523.5364 | 57.94 | 1 |
| KIDLGSSNSYINLVTVLENLFGCLGIGVAK | 1 | K1(di-Gl); C23(Carbamidomethyl) | 37 | 0.004374784 | n.d. | n.d. | n.d. | 3308.7418 | 3 | 1103.5854 | 120.04 | 1 |
| KNNEEASKAIGYVK | 2 | K8(di-Gl) | 37 | 0.003908018 | K8(di-Gl) | 70 | 6.75E-06 | 1664.8572 | 3 | 555.6239 | 24.18 | 1 |
| LGLPGDNYSEISVCGSSKK | 6 | C14(Carbamidomethyl); K19(di-Gl) | 74 | 7.85157E-07 | C14(Carbamidomethyl); K18(di-Gl) | 84 | 9.73E-23 | 2125.0242 | 2 | 1063.0157 | 47.74 | 1 |
| LGLPGDNYSEISVCGSSKKK | 3 | C14(Carbamidomethyl); K18(di-Gl); K19(di-Gl) | 24 | 0.088299159 | C14(Carbamidomethyl); K18(di-Gl); K19(di-Gl) | 60 | 3.87E-25 | 2367.1574 | 4 | 592.5448 | 41.06 | 1 |
| LGLPGDNYSEISVCGSSKKK | 3 | C14(Carbamidomethyl); K18(di-Gl) | 38 | 0.003364779 | C14(Carbamidomethyl); K18(di-Gl) | 119 | 3.87E-25 | 2253.1144 | 4 | 564.0341 | 41.18 | 1 |
| NNEEASKAIGYVK | 4 | K7(di-Gl) | 81 | 1.62913E-07 | K7(di-Gl) | 93 | 1.15E-08 | 1536.7672 | 2 | 768.8866 | 30.86 | 1 |
| AGFMAKEGLALEITELR | 5 | K6(di-Gl) | 90 | 1.90527E-08 | K6(di-Gl) | 103 | 3.30E-45 | 1963.0240 | 2 | 982.0156 | 67.84 | 2 |
| AIGYVKVSMDGVPYMR | 1 | K6(di-Gl) | 45 | 0.000617955 | K6(di-Gl) | 80 | 3.15E-08 | 1899.9382 | 3 | 633.9843 | 58.23 | 2 |
| KAGFMAKEGLALEITELR | 4 | K7(di-Gl) | 85 | 6.0528E-08 | K7(di-Gl) | 84 | 7.35E-09 | 2091.1244 | 4 | 523.5366 | 58.60 | 2 |
| KCEYIIIYEDKDR | 1 | K1(di-Gl); C2(Carbamidomethyl) | 20 | 0.185761867 | K1(di-Gl); C2(Carbamidomethyl) | 47 | 7.72E-12 | 1858.8969 | 3 | 620.3038 | 38.53 | 2 |
| LGLPGDNYSEISVCGSSKK | 8 | C14(Carbamidomethyl); K19(di-Gl) | 85 | 6.71362E-08 | C14(Carbamidomethyl); K19(di-Gl) | 57 | 2.06E-10 | 2125.0218 | 2 | 1063.0145 | 48.14 | 2 |
| LGLPGDNYSEISVCGSSKKK | 11 | C14(Carbamidomethyl); K19(di-Gl) | 39 | 0.002517425 | C14(Carbamidomethyl); K19(di-Gl) | 95 | 6.45E-14 | 2253.1163 | 3 | 751.7095 | 41.23 | 2 |
| LGLPGDNYSEISVCGSSKKK | 3 | C14(Carbamidomethyl); K18(LRGG) | 22 | 0.13211635 | C14(Carbamidomethyl); K18(LRGG); K19(di-Gl) | 63 | 6.45E-14 | 2522.3004 | 5 | 505.2659 | 38.40 | 2 |
| LGLPGDNYSEISVCGSSKKK | 9 | C14(Carbamidomethyl); K18(di-Gl); K20(di-Gl) | 43 | 0.000924606 | C14(Carbamidomethyl); K18(di-Gl); K19(di-Gl) | 95 | 6.45E-14 | 2367.1516 | 3 | 789.7233 | 41.15 | 2 |
| NNEEASKAIGYVK | 3 | K7(di-Gl) | 92 | 1.36131E-08 | K7(di-Gl) | 141 | 5.06E-16 | 1536.7632 | 2 | 768.8849 | 31.41 | 2 |
| AGFMAKEGLALEITELR | 6 | K6(di-Gl) | 84 | 8.78935E-08 | K6(di-Gl) | 84 | 1.36E-11 | 1963.0259 | 3 | 655.0135 | 69.77 | 3 |
| KAGFMAKEGLALEITELR | 4 | K7(di-Gl) | 72 | 1.27338E-06 | K7(di-Gl) | 81 | 1.30E-09 | 2091.1288 | 3 | 697.7151 | 58.42 | 3 |
| KNNEEASKAIGYVK | 2 | K8(di-Gl) | 30 | 0.02055685 | n.d. | n.d. | n.d. | 1664.8606 | 3 | 555.6251 | 24.30 | 3 |
| LGLPGDNYSEISVCGSSKK | 6 | C14(Carbamidomethyl); K18(di-Gl) | 83 | 1.01148E-07 | C14(Carbamidomethyl); K18(di-Gl) | 88 | 2.89E-13 | 2125.0206 | 3 | 709.0117 | 47.75 | 3 |
| LGLPGDNYSEISVCGSSKK | 2 | C14(Carbamidomethyl); K18(LRGG) | 42 | 0.00130004 | C14(Carbamidomethyl); K18(LRGG) | 34 | 2.89E-13 | 2394.2085 | 3 | 798.7410 | 43.56 | 3 |
| LGLPGDNYSEISVCGSSKKK | 6 | C14(Carbamidomethyl); K18(di-Gl) | 42 | 0.001180203 | C14(Carbamidomethyl); K18(di-Gl) | 63 | 7.78E-10 | 2253.1177 | 3 | 751.7108 | 41.33 | 3 |
| LGLPGDNYSEISVCGSSKKK | 3 | C14(Carbamidomethyl); K18(di-Gl); K19(di-Gl) | 21 | 0.170591178 | C14(Carbamidomethyl); K18(di-Gl); K19(di-Gl) | 63 | 7.78E-10 | 2367.1601 | 4 | 592.5455 | 41.13 | 3 |
| LGLPGDNYSEISVCGSSKKK | 3 | C14(Carbamidomethyl); K18(LRGG) | 21 | 0.146877939 | C14(Carbamidomethyl); K18(LRGG) | 17 | 7.78E-10 | 2522.3024 | 5 | 505.2663 | 37.97 | 3 |
| NNEEASKAIGYVK | 1 | K7(di-Gl) | 72 | 1.29705E-06 | K7(di-Gl) | 142 | 3.71E-37 | 1536.7665 | 3 | 512.9270 | 31.29 | 3 |
| NNEEASKAIGYVK | 1 | K7(LRGG) | 16 | 0.509279938 | K7(LRGG) | 58 | 3.71E-37 | 1805.9509 | 4 | 452.2432 | 29.27 | 3 |

*PEP: Posterior error probability
n.d. : not detected

## Supplementary Table 2. IAA19 ubiquitylated peptides identified in LC-MS analyses

### IAA19 w/o Auxin

| | | Mascot | | | MaxQuant | | | | | | | |
|---|---|---|---|---|---|---|---|---|---|---|---|---|
| Sequence | # PSMs | Modifications | IonScore | Exp Value | Modifications | Score | PEP | MH+ [Da] | Charge | m/z [Da] | RT | Exp |
| AGFMEKEGLGLEITELR | 1 | M4(Oxidation); K6(di-Gl) | 43 | 0.001016147 | M4(Oxidation); K6(di-Gl) | 56 | 1.40E-24 | 2023.0063 | 3 | 675.0070 | 59.26 | 1 |
| AGFMEKEGLGLEITELR | 1 | K6(di-Gl) | 53 | 9.93E-05 | K6(di-Gl) | 69 | 1.40E-24 | 2007.0161 | 3 | 669.6769 | 64.69 | 1 |
| EASTTKVGLGYVK | 1 | K6(LRGG) | 29 | 0.023656831 | K6(LRGG) | 20 | 0.00095458 | 1735.9674 | 4 | 434.7473 | 29.65 | 1 |
| KAGFMEKEGLGLEITELR | 2 | K7(di-Gl) | 51 | 0.000167478 | n.d. | n.d. | n.d. | 2135.1066 | 3 | 712.3737 | 55.77 | 1 |
| KMDLGSSQGYDDLAFALDK | 1 | K1(LRGG) | 66 | 5.31E-06 | K1(LRGG) | 81 | 1.75E-246 | 2457.2028 | 4 | 615.0562 | 57.59 | 1 |
| KMDLGSSQGYDDLAFALDK | 2 | K1(di-Gl); M2(Oxidation) | 74 | 8.09E-07 | K1(di-Gl); M2(Oxidation) | 67 | 1.75E-246 | 2204.0151 | 3 | 735.3432 | 58.72 | 1 |
| KMDLGSSQGYDDLAFALDK | 4 | K1(di-Gl) | 156 | 5.12E-15 | K1(di-Gl) | 190 | 1.75E-246 | 2188.0196 | 2 | 1094.5134 | 63.66 | 1 |
| LGLPGRDVAEKMMK | 1 | K11(di-Gl) | 16 | 0.558414348 | K11(di-Gl) | 50 | 2.86E-06 | 1658.8687 | 3 | 553.6278 | 43.92 | 1 |
| VGLGYVKVSMDGVPYLR | 1 | K7(di-Gl) | 64 | 8.63E-06 | K7(di-Gl) | 128 | 3.75E-31 | 1967.0354 | 3 | 656.3500 | 65.19 | 1 |
| VNDSPAAKSQVVGWPPVCSYR | 2 | K8(di-Gl); C18(Carbamidomethyl) | 60 | 1.87E-05 | K8(di-Gl); C18(Carbamidomethyl) | 75 | 1.64E-13 | 2431.1748 | 3 | 811.0631 | 51.52 | 1 |
| AGFMEKEGLGLEITELR | 1 | M4(Oxidation); K6(di-Gl) | 33 | 0.009659543 | M4(Oxidation); K6(di-Gl) | 29 | 9.57E-80 | 2023.0147 | 3 | 675.0098 | 59.56 | 2 |
| AGFMEKEGLGLEITELR | 2 | K6(di-Gl) | 53 | 9.79E-05 | K6(di-Gl) | 62 | 9.57E-80 | 2007.0192 | 3 | 669.6779 | 65.13 | 2 |
| DVAEKMMK | 4 | K5(di-Gl); M7(Oxidation) | 16 | 0.497687311 | K5(di-Gl); M7(Oxidation) | 79 | 6.03E-07 | 1081.5008 | 2 | 541.2540 | 16.86 | 2 |
| DVAEKMMKK | 2 | K5(di-Gl); K8(di-Gl) | 20 | 0.190966227 | K5(di-Gl); K8(di-Gl) | 52 | 0.00067398 | 1307.6402 | 2 | 654.3237 | 20.18 | 2 |
| EASTTKVGLGYVK | 2 | K6(LRGG) | 29 | 0.02666592 | K6(LRGG) | 40 | 4.98E-06 | 1735.9709 | 4 | 434.7476 | 29.79 | 2 |
| EASTTKVGLGYVK | 1 | K6(di-Gl) | 14 | 0.751547732 | K6(di-Gl) | 32 | 4.98E-06 | 1466.7848 | 3 | 489.5998 | 33.28 | 2 |
| GIGVALKDGDNCEYVTIYEDK | 1 | K7(di-Gl); C12(Carbamidomethyl) | 85 | 6.68E-08 | K7(di-Gl); C12(Carbamidomethyl) | 54 | 4.69E-40 | 2473.1561 | 3 | 825.0569 | 58.68 | 2 |
| KAGFMEKEGLGLEITELR | 2 | K1(di-Gl); K7(di-Gl) | 59 | 2.58E-05 | K1(di-Gl); K7(di-Gl) | 68 | 3.73E-44 | 2249.1555 | 3 | 750.3900 | 55.76 | 2 |
| KMDLGSSQGYDDLAFALDK | 1 | K1(LRGG) | 64 | 8.41E-06 | K1(LRGG) | 87 | 7.38E-113 | 2457.2072 | 4 | 615.0573 | 58.01 | 2 |
| KMDLGSSQGYDDLAFALDK | 3 | K1(di-Gl); M2(Oxidation) | 94 | 7.89E-09 | K1(di-Gl); M2(Oxidation) | 95 | 7.38E-113 | 2204.0151 | 3 | 735.3432 | 59.09 | 2 |
| KMDLGSSQGYDDLAFALDK | 4 | K1(di-Gl) | 134 | 8.43E-13 | K1(di-Gl) | 148 | 7.38E-113 | 2188.0218 | 2 | 1094.5145 | 64.18 | 2 |
| LGLPGRDVAEKMMK | 3 | K11(di-Gl) | 19 | 0.250009533 | K11(di-Gl) | 82 | 1.44E-10 | 1658.8703 | 4 | 415.4730 | 44.53 | 2 |
| NSCKEASTTKVGLGYVK | 1 | C3(Carbamidomethyl); K4(di-Gl); K10(di-Gl) | 72 | 1.16E-06 | n.d. | n.d. | n.d. | 2070.0320 | 3 | 690.6822 | 27.69 | 2 |
| NSCKEASTTKVGLGYVK | 2 | C3(Carbamidomethyl); K10(di-Gl) | 24 | 0.082216043 | n.d. | n.d. | n.d. | 1955.9884 | 4 | 489.7526 | 26.8 | 2 |
| VGLGYVKVSMDGVPYLR | 1 | K7(LRGG) | 16 | 0.463400575 | n.d. | n.d. | n.d. | 2236.2211 | 4 | 559.8107 | 59.82 | 2 |
| VGLGYVKVSMDGVPYLR | 1 | K7(di-Gl) | 57 | 3.97E-05 | K7(di-Gl) | 97 | 1.26E-18 | 1967.0398 | 3 | 656.3514 | 65.51 | 2 |
| VNDSPAAKSQVVGWPPVCSYR | 2 | K8(LRGG); C18(Carbamidomethyl) | 32 | 0.013550539 | n.d. | n.d. | n.d. | 2700.3656 | 4 | 675.8469 | 46.98 | 2 |
| VNDSPAAKSQVVGWPPVCSYR | 3 | K8(di-Gl); C18(Carbamidomethyl) | 60 | 1.95E-05 | K8(di-Gl); C18(Carbamidomethyl) | 82 | 1.65E-17 | 2431.1762 | 3 | 811.0636 | 51.56 | 2 |
| AGFMEKEGLGLEITELR | 2 | K6(di-Gl) | 102 | 1.15E-09 | K6(di-Gl) | 139 | 4.29E-175 | 2007.0162 | 2 | 1004.0117 | 65.28 | 3 |
| EASTTKVGLGYVK | 1 | K6(di-Gl) | 34 | 0.007942488 | K6(di-Gl) | 27 | 1.35E-09 | 1466.7843 | 3 | 489.5996 | 32.31 | 3 |
| EASTTKVGLGYVK | 1 | K6(LRGG) | 15 | 0.587430604 | n.d. | n.d. | n.d. | 1735.9710 | 4 | 434.7482 | 29.98 | 3 |
| KMDLGSSQGYDDLAFALDK | 2 | K1(di-Gl) | 136 | 4.99E-13 | K1(di-Gl) | 165 | 8.41E-97 | 2188.0228 | 2 | 1094.5150 | 64.22 | 3 |
| KMDLGSSQGYDDLAFALDK | 1 | K1(LRGG) | 47 | 0.000386328 | K1(LRGG) | 116 | 8.41E-97 | 2457.2079 | 4 | 615.0574 | 58.04 | 3 |
| LGLPGRDVAEKMMK | 1 | K11(di-Gl) | 16 | 0.534510903 | K11(di-Gl) | 80 | 3.17E-06 | 1658.8698 | 3 | 553.6281 | 44.54 | 3 |
| NSCKEASTTKVGLGYVK | 2 | C3(Carbamidomethyl); K10(di-Gl) | 29 | 0.022592098 | n.d. | n.d. | n.d. | 1955.9857 | 4 | 489.7519 | 26.69 | 3 |
| VGLGYVKVSMDGVPYLR | 1 | K7(di-Gl) | 45 | 0.000612289 | K7(di-Gl) | 74 | 6.70E-09 | 1967.0350 | 3 | 656.3499 | 65.59 | 3 |
| VNDSPAAKSQVVGWPPVCSYR | 2 | K8(di-Gl); C18(Carbamidomethyl) | 58 | 3.20E-05 | K8(di-Gl); C18(Carbamidomethyl) | 110 | 1.82E-22 | 2431.1788 | 3 | 811.0645 | 52.07 | 3 |

### IAA19 with 4 μM IAA

| | | Mascot | | | MaxQuant | | | | | | | |
|---|---|---|---|---|---|---|---|---|---|---|---|---|
| Sequence | # PSMs | Modifications | IonScore | Exp Value | Modifications | Score | PEP | MH+ [Da] | Charge | m/z [Da] | RT | Exp |
| AGFMEKEGLGLEITELR | 1 | M4(Oxidation); K6(di-Gl) | 73 | 1.10E-06 | M4(Oxidation); K6(di-Gl) | 58 | 1.13E-50 | 2023.0136 | 3 | 675.0094 | 58.99 | 1 |
| AGFMEKEGLGLEITELR | 2 | K6(LRGG) | 23 | 0.111932594 | K6(LRGG) | 36 | 1.13E-50 | 2276.2140 | 4 | 569.8090 | 58.88 | 1 |
| AGFMEKEGLGLEITELR | 3 | K6(di-Gl) | 84 | 7.55E-08 | K6(di-Gl) | 92 | 1.13E-50 | 2007.0154 | 2 | 1004.0114 | 64.48 | 1 |
| DVAEKMMK | 8 | M6(Oxidation); K8(di-Gl) | 22 | 0.115068531 | n.d. | n.d. | n.d. | 1081.4987 | 2 | 541.2537 | 17.58 | 1 |
| EASTTKVGLGYVK | 2 | K6(LRGG) | 26 | 0.053574308 | K6(LRGG) | 27 | 8.78E-07 | 1735.9676 | 4 | 434.7473 | 29.53 | 1 |
| GIGVALKDGDNCEYVTIYEDK | 3 | K7(di-Gl); C12(Carbamidomethyl) | 104 | 7.87E-10 | K7(di-Gl); C12(Carbamidomethyl) | 38 | 2.63E-41 | 2473.1471 | 3 | 825.0539 | 57.72 | 1 |
| KAGFMEKEGLGLEITELR | 4 | K7(di-Gl) | 65 | 6.44E-06 | K7(di-Gl) | 113 | 2.26E-40 | 2135.1125 | 4 | 534.5336 | 55.69 | 1 |
| KAGFMEKEGLGLEITELR | 2 | K1(di-Gl); K7(di-Gl) | 100 | 1.81E-09 | K1(di-Gl); K7(di-Gl) | 142 | 2.26E-40 | 2249.1522 | 3 | 750.3889 | 55.6 | 1 |
| KMDLGSSQGYDDLAFALDK | 1 | K1(LRGG) | 59 | 2.71E-05 | K1(LRGG) | 100 | 2.01E-194 | 2457.2045 | 4 | 615.0566 | 57.49 | 1 |
| KMDLGSSQGYDDLAFALDK | 6 | K1(di-Gl); M2(Oxidation) | 94 | 7.13E-09 | K1(di-Gl); M2(Oxidation) | 79 | 2.01E-194 | 2204.0136 | 3 | 735.3427 | 58.56 | 1 |
| KMDLGSSQGYDDLAFALDK | 5 | K1(di-Gl) | 156 | 5.03E-15 | K1(di-Gl) | 204 | 2.01E-194 | 2188.0203 | 2 | 1094.5138 | 63.62 | 1 |
| NSCKEASTTKVGLGYVK | 2 | C3(Carbamidomethyl); K4(di-Gl); K10(di-Gl) | 46 | 0.000493124 | C3(Carbamidomethyl); K4(di-Gl); K10(di-Gl) | 57 | 1.03E-06 | 2070.0229 | 3 | 690.6791 | 27.3 | 1 |
| VGLGYVKVSMDGVPYLR | 1 | K7(di-Gl); M10(Oxidation) | 26 | 0.053945667 | K7(di-Gl); M10(Oxidation) | 24 | 1.34E-39 | 1983.0351 | 3 | 661.6832 | 58.18 | 1 |

# Supplementary Table 2 continued. IAA19 ubiquitylated peptides identified in LC-MS analyses

## IAA19 with 4 µM IAA

| | | Mascot | | | MaxQuant | | | | | | | |
|---|---|---|---|---|---|---|---|---|---|---|---|---|
| Sequence | # PSMs | Modifications | IonScore | Exp Value | Modifications | Score | PEP | MH+ [Da] | Charge | m/z [Da] | RT | Exp |
| VGLGYVKVSMDGVPYLR | 2 | K7(di-Gl) | 88 | 3.03E-08 | K7(di-Gl) | 142 | 1.34E-39 | 1967.0392 | 2 | 984.0233 | 64.91 | 1 |
| VNDSPAAKSQVVGWPPVCSYR | 5 | K8(di-Gl); C18(Carbamidomethyl) | 66 | 4.61E-06 | K8(di-Gl); C18(Carbamidomethyl) | 89 | 1.64E-17 | 2431.1762 | 3 | 811.0636 | 51.35 | 1 |
| AGFMEKEGLGLEITELR | 1 | K6(di-Gl) | 62 | 1.39E-05 | K6(di-Gl) | 114 | 2.15E-113 | 2007.0187 | 3 | 669.6777 | 65.17 | 2 |
| EASTTKVGLGYVK | 2 | K6(di-Gl) | 45 | 0.00058608 | K6(di-Gl) | 39 | 4.04E-07 | 1466.7840 | 3 | 489.5995 | 32.11 | 2 |
| EASTTKVGLGYVK | 2 | K6(LRGG) | 26 | 0.051045395 | K6(LRGG) | 26 | 4.04E-07 | 1735.9694 | 4 | 434.7478 | 29.75 | 2 |
| GIGVALKDGDNCEYVTIYEDK | 1 | K7(di-Gl); C12(Carbamidomethyl) | 62 | 1.27E-05 | K7(di-Gl); C12(Carbamidomethyl) | 37 | 3.63E-104 | 2473.1556 | 3 | 825.0567 | 58.26 | 2 |
| KMDLGSSQGYDDLAFALDK | 2 | K1(di-Gl) | 117 | 3.89E-11 | K1(di-Gl) | 194 | 2.38E-114 | 2188.0218 | 2 | 1094.5145 | 64.21 | 2 |
| KMDLGSSQGYDDLAFALDK | 2 | K1(di-Gl); M2(Oxidation) | 93 | 8.95E-09 | K1(di-Gl); M2(Oxidation) | 101 | 2.38E-114 | 2204.0167 | 3 | 735.3438 | 59.03 | 2 |
| KMDLGSSQGYDDLAFALDK | 2 | K1(LRGG) | 84 | 8.15E-08 | K1(LRGG) | 122 | 2.38E-114 | 2457.2045 | 3 | 819.7397 | 58.12 | 2 |
| LGLPGRDVAEKMMK | 2 | K11(di-Gl) | 13 | 0.988454239 | K11(di-Gl) | 52 | 0.00017596 | 1658.8719 | 2 | 829.9396 | 44.6 | 2 |
| NSCKEASTTKVGLGYVK | 5 | C3(Carbamidomethyl); K10(di-Gl) | 55 | 6.81E-05 | C3(Carbamidomethyl); K10(di-Gl) | 80 | 8.73E-10 | 1955.9851 | 4 | 489.7523 | 26.77 | 2 |
| NSCKEASTTKVGLGYVK | 1 | C3(Carbamidomethyl); K4(di-Gl); K10(di-Gl) | 14 | 0.79059956 | C3(Carbamidomethyl); K4(di-Gl); K10(di-Gl) | 26 | 8.73E-10 | 2070.0285 | 4 | 518.2626 | 27.89 | 2 |
| VGLGYVKVSMDGVPYLR | 1 | K7(di-Gl) | 60 | 2.00E-05 | K7(di-Gl) | 105 | 5.87E-17 | 1967.0425 | 3 | 656.3524 | 65.5 | 2 |
| VNDSPAAKSQVVGWPPVCSYR | 2 | K8(di-Gl); C18(Carbamidomethyl) | 60 | 1.91E-05 | K8(di-Gl); C18(Carbamidomethyl) | 120 | 8.62E-28 | 2431.1806 | 3 | 811.0651 | 51.98 | 2 |
| VNDSPAAKSQVVGWPPVCSYR | 1 | K8(LRGG); C18(Carbamidomethyl) | 15 | 0.608074188 | K8(LRGG); C18(Carbamidomethyl) | 18 | 8.62E-28 | 2700.3666 | 4 | 675.8471 | 46.77 | 2 |
| AGFMEKEGLGLEITELR | 1 | K6(di-Gl) | 60 | 1.79E-05 | K6(di-Gl) | 89 | 1.19E-96 | 2007.0157 | 3 | 669.6768 | 65.49 | 3 |
| EASTTKVGLGYVK | 1 | K6(di-Gl) | 58 | 3.31E-05 | K6(di-Gl) | 39 | 1.12E-05 | 1466.7834 | 3 | 489.5993 | 32.81 | 3 |
| EASTTKVGLGYVK | 2 | K6(LRGG) | 24 | 0.074637411 | K6(LRGG) | 28 | 3.94E-28 | 1735.9707 | 4 | 434.7481 | 30.39 | 3 |
| GIGVALKDGDNCEYVTIYEDK | 1 | K7(di-Gl); C12(Carbamidomethyl) | 65 | 7.08E-06 | K7(di-Gl); C12(Carbamidomethyl) | 48 | 1.48E-63 | 2473.1545 | 3 | 825.0563 | 58.65 | 3 |
| KMDLGSSQGYDDLAFALDK | 2 | K1(di-Gl) | 130 | 1.88E-12 | K1(di-Gl) | 191 | 5.11E-115 | 2188.0247 | 2 | 1094.5160 | 64.52 | 3 |
| KMDLGSSQGYDDLAFALDK | 1 | K1(di-Gl); M2(Oxidation) | 66 | 4.93E-06 | K1(di-Gl); M2(Oxidation) | 71 | 5.11E-115 | 2204.0083 | 3 | 735.3409 | 59.74 | 3 |
| NSCKEASTTKVGLGYVK | 4 | C3(Carbamidomethyl); K10(di-Gl) | 37 | 0.003980674 | C3(Carbamidomethyl); K10(di-Gl) | 45 | 0.00046473 | 1955.9811 | 3 | 652.6652 | 27.23 | 3 |
| NSCKEASTTKVGLGYVK | 1 | C3(Carbamidomethyl); K4(di-Gl); K10(di-Gl) | 36 | 0.004644688 | C3(Carbamidomethyl); K4(di-Gl); K10(di-Gl) | 31 | 0.00046473 | 2070.0324 | 3 | 690.6823 | 28.22 | 3 |
| VGLGYVKVSMDGVPYLR | 1 | K7(di-Gl) | 63 | 1.01E-05 | K7(di-Gl) | 93 | 1.22E-11 | 1967.0387 | 3 | 656.3511 | 65.95 | 3 |
| VNDSPAAKSQVVGWPPVCSYR | 3 | K8(di-Gl); C18(Carbamidomethyl) | 60 | 2.04E-05 | K8(di-Gl); C18(Carbamidomethyl) | 91 | 8.32E-15 | 2431.1812 | 3 | 811.0653 | 52.53 | 3 |

# Supplementary Note 1

## Population genetic and gene expression analyses

AtGenExpress[1] (http://jsp.weigelworld.org/AtGenExpress/resources/), and *Arabidopsis* eFP[2] (http://www.bar.utoronto.ca/) browsers were used to retrieve and compare *A. thaliana* expression profiles for *IAA6* and *IAA19* in different natural accessions[3], and developmental stages, as well as different tissues including: root cells types[4, 5], microgametogenesis[6], embryo development[7], flowers[3], xylem & cork[8], guard & mesophyll cells[9], stem epidermis[10], stigma & ovaries[11], pollen germination[12], shot apical[13], trichomes[14, 15].

## References


1. Schmid, M. *et al.* A gene expression map of Arabidopsis thaliana development. *Nat Genet* **37**, 501-506 (2005).
2. Winter, D. *et al.* An "Electronic Fluorescent Pictograph" browser for exploring and analyzing large-scale biological data sets. *PLoS One* **2**, e718 (2007).
3. Lempe, J. *et al.* Diversity of flowering responses in wild Arabidopsis thaliana strains. *PLoS Genet* **1**, 109-118 (2005).
4. Birnbaum, K. *et al.* A gene expression map of the Arabidopsis root. *Science* **302**, 1956-1960 (2003).
5. Nawy, T. *et al.* Transcriptional profile of the Arabidopsis root quiescent center. *Plant Cell* **17**, 1908-1925 (2005).
6. Honys, D. & Twell, D. Transcriptome analysis of haploid male gametophyte development in Arabidopsis. *Genome Biol* **5**, R85 (2004).
7. Casson, S., Spencer, M., Walker, K. & Lindsey, K. Laser capture microdissection for the analysis of gene expression during embryogenesis of Arabidopsis. *Plant J* **42**, 111-123 (2005).
8. Zhao, C., Craig, J.C., Petzold, H.E., Dickerman, A.W. & Beers, E.P. The xylem and phloem transcriptomes from secondary tissues of the Arabidopsis root-hypocotyl. *Plant Physiol* **138**, 803-818 (2005).
9. Yang, Y., Costa, A., Leonhardt, N., Siegel, R.S. & Schroeder, J.I. Isolation of a strong Arabidopsis guard cell promoter and its potential as a research tool. *Plant Methods* **4**, 6 (2008).
10. Suh, M.C. *et al.* Cuticular lipid composition, surface structure, and gene expression in Arabidopsis stem epidermis. *Plant Physiol* **139**, 1649-1665 (2005).

11. Swanson, R., Clark, T. & Preuss, D. Expression profiling of Arabidopsis stigma tissue identifies stigma-specific genes. *Sexual Plant Reproduction* **18**, 163-171 (2005).
12. Qin, Y. *et al.* Penetration of the stigma and style elicits a novel transcriptome in pollen tubes, pointing to genes critical for growth in a pistil. *PLoS Genet* **5**, e1000621 (2009).
13. Yadav, R.K., Girke, T., Pasala, S., Xie, M. & Reddy, G.V. Gene expression map of the Arabidopsis shoot apical meristem stem cell niche. *Proc Natl Acad Sci U S A* **106**, 4941-4946 (2009).
14. Gilding, E.K. & Marks, M.D. Analysis of purified glabra3-shapeshifter trichomes reveals a role for NOECK in regulating early trichome morphogenic events. *Plant J* **64**, 304-317 (2010).
15. Marks, M.D., Wenger, J.P., Gilding, E., Jilk, R. & Dixon, R.A. Transcriptome analysis of Arabidopsis wild-type and gl3-sst sim trichomes identifies four additional genes required for trichome development. *Mol Plant* **2**, 803-822 (2009).
