# Supplementary material for: Variation in auxin sensing guides AUX/IAA transcriptional repressor ubiquitylation and destruction
Source: Nat Commun. 2017 Jun 7;8:15706. doi: 10.1038/ncomms15706 (PMC5467235; doi:10.1038/ncomms15706)

Supplementary Data 1

Sequence alignment of 29 *A. thaliana* AUX/IAA proteins generated for the phylogenetic tree in **Supplementary Fig. 1** with MAFFT using the L-INS-i option (Katoh, Kuma et al. 2005). Java multiple alignment editor, Jalview (2009) (http://www.jalview.org) using default settings was used for visualization. IAA6 (AT1G52830; green) and IAA19 (AT3G15540; blue) share 61.4% identity with 121 of 197 residues overlapping. IAA6 consists of 9.5% (18) Lys, 9.6% (20) Ser, 2.6% (5) Cys and Thr each, 3.7% (7) Tyr residues. IAA19 consists of 8.6% (17) Lys and Ser each, 2.5% (5) Cys, 3.6% (7) Thr, 3.0% (6) Tyr residues. Black residues and bars at the bottom represent consensus, while yellow bars chart indicates conservation of the total alignment with less than 25% gaps. The following AUX/IAA consensus domain structures are depicted: i) EAR-motif containing domain I (DI) (orange lines), which recruits TOPLESS/TOPLESS RELATED corepressors, ii) core or primary degron (red) and degron flanking regions (rate motifs) (blue) in domain II (DII), which interact directly with TIR1/AFBs and are required for auxin-dependent co-repressor assembly, and iii) C-terminal PB1 domains (DIII-IV) composed of three helices and five mixed-stranded β-sheets, which adopt an ubiquitin-like β grasp fold and mediate homo- and heterotypic interactions between AUX/IAAs and ARFs. Consensus sequences of a single and a bipartite nuclear localization signal (NLS) (orange lines) are also shown.

Supplementary Data 1

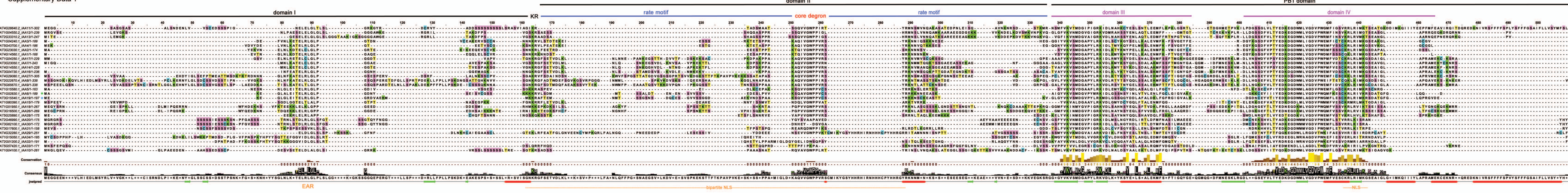

Supplement: Supplementary Data 1 — Sequence alignment of 29 A. thaliana AUX/IAA proteins generated for the phylogenetic tree in Supplementary Fig. 1. [file ncomms15706-s2.pdf]
