# Supplementary material for: Variation in auxin sensing guides AUX/IAA transcriptional repressor ubiquitylation and destruction
Source: Nat Commun. 2017 Jun 7;8:15706. doi: 10.1038/ncomms15706 (PMC5467235; doi:10.1038/ncomms15706)

Supplementary Data 2

Multiple sequence alignments of AUX/IAAs from 81 *A. thaliana* accessions and *A. halleri, A. lyrata, or C. rubella* were generated with MAFFT and used for sliding window *dN/dS* analysis. Figshare https://figshare.com/s/6e202a97eb8034bbb1d9 for fasta files.

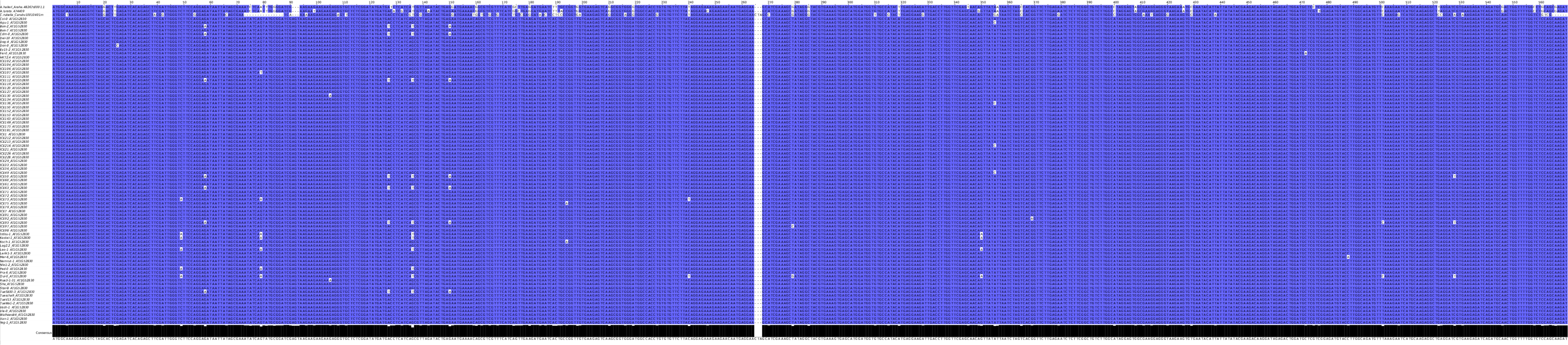

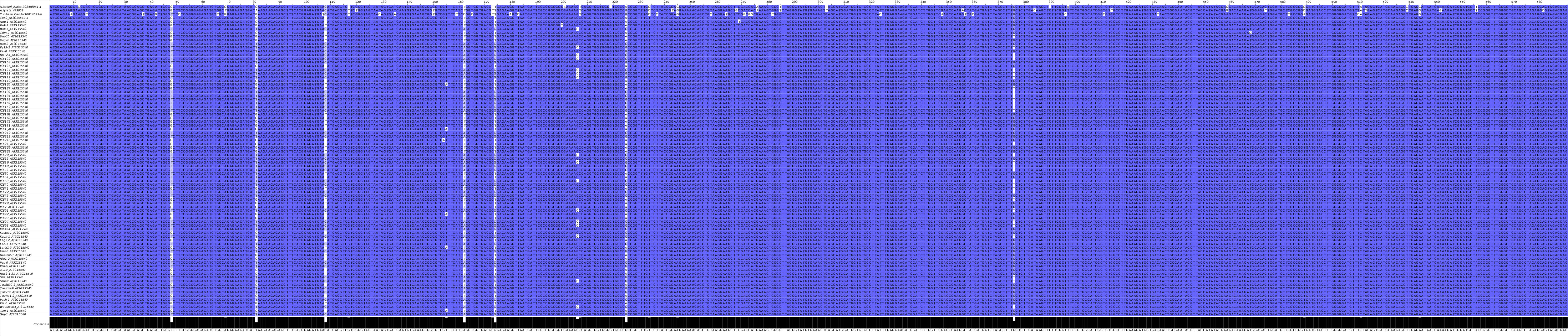

Supplement: Supplementary Data 2 — Multiple sequence alignments of AUX/IAAs from 81 A. thaliana accessions and A. halleri, A. lyrata, and C. rubella [file ncomms15706-s3.pdf]
